# A new extension of Burr-Hatke exponential distribution with engineering and biomedical applications

**DOI:** 10.1016/j.heliyon.2024.e38293

**Published:** 2024-09-26

**Authors:** Kizito E. Anyiam, Fatimah M. Alghamdi, Chrysogonus C. Nwaigwe, Hassan M. Aljohani, Okechukwu J. Obulezi

**Affiliations:** a Department of Statistics, Federal University of Technology, Owerri, Nigeria; b Department of Mathematical Sciences, College of Science, Princess Nuorah bint Abdulrahman University, P. O. Box 84428, Riyadh 11671, Saudi Arabia; c Department of Mathematics and Statistics, College of Science, Taif University, P. O. Box 11099, Taif 21944, Saudi Arabia; d Department of Statistics, Faculty of Physical Sciences, Nnamdi Azikiwe University, P. O. Box 5025, Awka, Nigeria

**Keywords:** Topp-Leone Burr Hakte exponential distribution, Stress strength reliability, Maximum likelihood estimation, Bayesian estimation, Confidence intervals, Parametric regression, Censored data

## Abstract

In this study, the Topp-Leone family of distribution approach was used to modify the Burr Hatke Exponential distribution to provide adequate fits for some engineering and health data which previous existing distributions in the family of Burr Hatke Exponential have failed to do. The new distribution improves the robustness of Burr Hatke Exponential distribution by making it capable of modeling emerging new world complex data with varying features, possesses greater capacity and flexibility to model lifetime data, has better goodness of fit. Some mathematical properties of the derived distribution such as quantile function, moments, order statistics, entropies, etc were obtained and discussed. Some non-Bayesian estimation approaches like maximum likelihood estimation (ML), maximum Product spacing estimation (MPSE), Least squares estimation (LS), weighted least squares estimation (WLS), Cramer-von-Mises estimation (CVM), Anderson-Darling estimation (AD), and right-tailed Anderson Darling estimation (RTAD), as well as Bayesian method under independent gamma priors were adopted to estimate the parameters of the model and the various methods proved efficient. From the simulation results, the bias and root mean squared error for the parameters are relatively small and the become smaller as the sample size becomes larger. This shows convenience and improved estimation accuracy. We further constructed a regression model using the proposed distribution. Extensive simulation studies were used to determine the efficiency of the method in both the estimation of its parameters and that of the regression model. The TL-BHE regression was fitted on censored CD4 count data of HIV/AIDs patients. The competitiveness, applicability, and usefulness of the model were demonstrated using datasets and the results validate the theoretical findings. The results indicate that the TL-BHE distribution achieved better results than the baseline distribution and other variants of the classical distributions.

## 1 Introduction

Making an unsuitable choice of distribution in modeling data in engineering and medical sciences may lead to wrong inferences with critical consequences, especially in precision engineering and disease control. Moreover, parametric distributions are important in modeling lifetime data from engineering and medicine. Recently, many new or modified distributions and associated regression models have been advanced to improve the quality and accuracy of statistical outputs. Since the quality of the statistical analysis depends heavily on the assumed model, various statisticians attempt to define new families of univariate distributions that extend some well-known distributions.

Topp-Leone distribution pioneered by Topp and Leone [51] has proved to possess some potential that made it to be adopted by Sangsanit and Bodhisuwan [45], Al-Shomrani et al. [47]to develop the Topp-Leone -G (TL-G) family of distributions. Advancing the development of families of distributions has revolutionized probability theory and methods leading to the improvement in the parent distribution. Other works in which TL-G family have played significant roles include, Topp-Leone Exponentiated Lomax (TL-EL) distribution by Sule et al. [48], Topp-Leone Lomax (TLLo) distribution by Oguntunde et al. [38], Topp-Leone Marshall Olkin-Weibull (TL-MOW) distribution by Ahmed et al. [4], Topp-Leone Dagum (TL-D) distribution by Rasheed [40], The Topp-Leone odd log-logistic (TLOL-G) family of distributions by Brito et al. [11], Topp-Leone generalized odd log-logistic (TL=GOL) distribution by Korkmaz et al. [28]. See Hassan et al. [42], Elgarhy et al. [21], Hassan and Almetwally [25], Metwally et al. [32] for other useful models from the TL-G family.

Burr-Hatke Generator (BH-G) of distributions has been getting attention in the literature since its development by [26]. Some interesting variants include the two-parameter BH distribution by [3], exponentiated Burr-Hatke (EBH) distribution with its discrete version by [33], logarithmic Burr-Hatke (LBH) distribution by [1], Chen Burr-Hatke exponential (CH-BH) by [2] which very mathematically tractable to lend itself to parametric regression transformation and biomedical applications. Others include inverse power Burr-Hatke (IPBH) distribution by [29], alpha power inverse power Burr-Hatke (APIPBH) distribution by [6], the discrete extension of BH distribution using generalized hypergeometric function by [7], Unit Burr-Hatke (UBH) distribution with a new quantile regression model by [43], and weighted Burr-Hatke (WBH) distribution by [5], logistic exponential distribution under progressive type-I hybrid censored sample by Dutta and Kayal [16], parametric inferences using dependent competing risks data with partially observed failure causes from MOBK distribution under unified hybrid censoring Dutta et al. [17], Gumbel type-II distribution under simple step-stress life test using type-II censoring Dutta et al. [20], a general family of distributions based on simple step-stress life test using TRV model under type II censoring Dutta et al. [19], logistic-exponential distribution using improved adaptive type-II progressively censored data Dutta et al. [15], Half Logistic Generalized Rayleigh Distribution for Modeling Hydrological Data Ogunde et al. [37], and Kumaraswamy-G family of distributions under unified progressive hybrid censoring with partially observed competing risks data Dutta et al. [18].

The spotlight of this study is the Burr Hatke Exponential (BHE) distribution, a one-parameter distribution introduced by Yadav et al. [55] with only right-skewed and decreasing failure rate function characteristics. The BH distribution has proven to be useful because of the role it has played in modeling lifetime datasets in reliability engineering, biological sciences, health sciences, computer sciences, biomedical, and risk management. A drawback is that BHE distribution cannot provide adequate fits to some real-life data. This article is therefore motivated by the need;

• To improve the robustness of BHE distribution by making it capable of modeling emerging new world complex data with varying features. This is achieved through the uniqueness of the functional form of the new distribution.
• To develop an extension of BHE distribution with greater capacity and flexibility to model lifetime data.
• To create a new version of BHE distribution with a better goodness of fit.
• To add a parameter on BHE, responsible for the shape which inadvertently increases the applicability of the TL-BHE in real data sets.

The rest of this article are organized as follows; Section 2 discusses the specification of the new member of the TL-G family. Section 3 provides the mixture representations and properties of the TL-BHE distribution. In section 4, classical non-Bayesian estimation approaches to parameter estimation like maximum likelihood estimation(ML), maximum Product spacing estimation (MPSE), Least squares estimation (LS), weighted least squares estimation (WLS), Cramer-von-Mises estimation (CVM), Anderson-Darling estimation (AD) and right-tailed Anderson Darling estimation (RTAD) are presented. Section 5 deals with the Bayesian estimation approach. In section 6, the TL-BHE parametric regression is discussed. In section 7, application to real-life data was carried out in section 8. Discussion and conclusion come up in section 9.

## 2 The TL-BHE distribution

Given the one parameter Burr Hakte Exponential (B-HE) distribution proposed by Yadav et al. [55] with the cumulative distribution function (cdf)

(1) $$G(x;\pi )=1-\frac{e^{-\pi x}}{1+\pi x},\;x>0,\;\pi >0,$$

with probability density function (pdf) given as

(2) $$g(x;\pi )=\pi e^{-\pi x}\frac{2+\pi x}{{\left(1+\pi x\right)}^{2}},$$

where *π* is the scale parameter adopted from the classical exponential distribution during the formation of the Burr-Hatke exponential distribution. Using the method introduced by Rasheed [40] the cdf of the TL-G family of distributions which introduces a shape parameter is given as

(3) $$F(x;\alpha ,\Psi )={\left[1-{\left(1-G(x)\right)}^{2}\right]}^{\alpha },$$

with pdf given as

(4) $$f(x;\alpha ,\Psi )=2\alpha g(x;\Psi )\overset{¯}{G}(x;\Psi ){\left[1-{\left(\overset{¯}{G}(x;\Psi )\right)}^{2}\right]}^{\alpha -1}.$$

Ψ is the vector of parameters of the baseline distribution with cdf and pdf respectively given as G(x;Ψ) and g(x;Ψ). Hence the cdf of the proposed Topp-Leone Burr Hatke Exponential (TL-BHE) distribution by substituting equation (1) into (3) and equations (1) and (2) into equation (4).

(5) $$F(x)={\left[1-{\left(\frac{e^{-\pi x}}{1+\pi x}\right)}^{2}\right]}^{\alpha },x>0,\pi >0,\alpha >0,$$

and the corresponding pdf and hazard rate function h(x) given in equations (6) and (7) respectively.

(6) $$f(x)=2a\pi e^{-2\pi x}\frac{\left(2+\pi x\right)}{{\left(1+\pi x\right)}^{3}}{\left[1-{\left(\frac{e^{-\pi x}}{1+\pi x}\right)}^{2}\right]}^{\alpha -1}.$$

and

(7) $$h(x)=\frac{2\alpha \pi e^{-2\pi x}\frac{2+\pi x}{{\left(1+\pi x\right)}^{3}}{\left[1-{\left(\frac{e^{-\pi x}}{1+\pi x}\right)}^{2}\right]}^{\alpha -1}}{1-{\left[1-{\left(\frac{e^{-\pi x}}{1+\pi x}\right)}^{2}\right]}^{\alpha }}$$

The quantile function which is very important for the characterization of the TL-BHE distribution, especially for generating random numbers is $Q(u)=F^{-1}(u)$ of X is derived in equation (8)

(8) $$u_{q}=\frac{1}{\pi }[W(e{\left({\left(1-u^{\frac{1}{\alpha }}\right)}^{\frac{1}{2}}\right)}^{-1}-1)]$$

where *W* is the Lambert W function (Corless et al. [13]), e is the inverse of the natural log function, u follows the uniform distribution on the interval (0,1), and $F^{-1}(.)$ is the inverse of F(.). Specifically, Q(0.25),Q(0.5), and Q(0.75) are the first, second, and third quartiles respectively. The quantile function can assist us in formalizing the peakedness and tail behavior of TL-BHE distribution. It is not difficult to show that the probability density function in Equation (6) is valid following necessary conditions.

**Figure 1:**
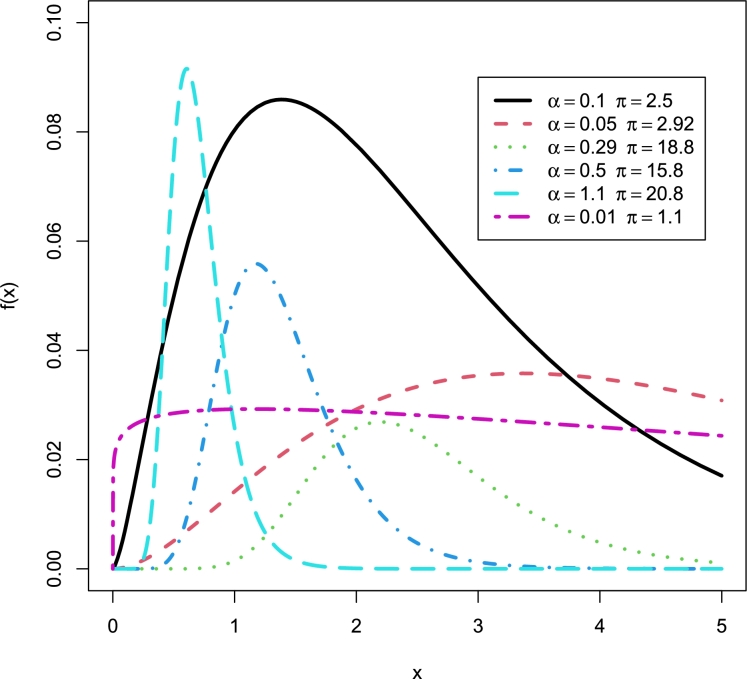
pdf of TL-HBE (*π*,*α*).

**Figure 2:**
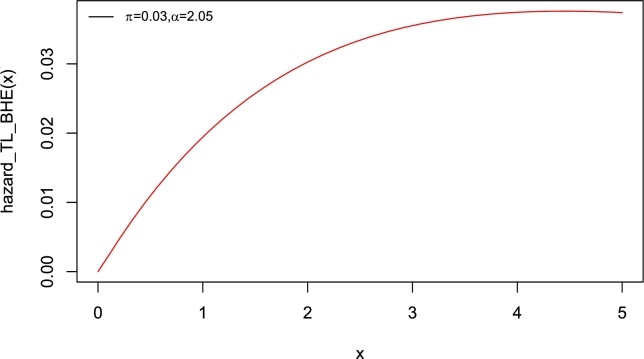
Hazard function of TL-HBE (*π*,*α*).

**Figure 3:**
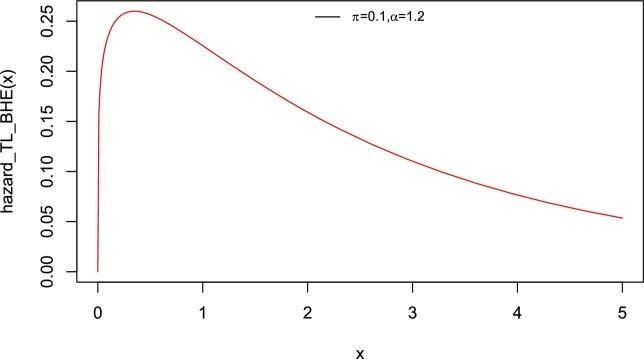
Hazard function of TL-HBE (*π*,*α*).

**Figure 4:**
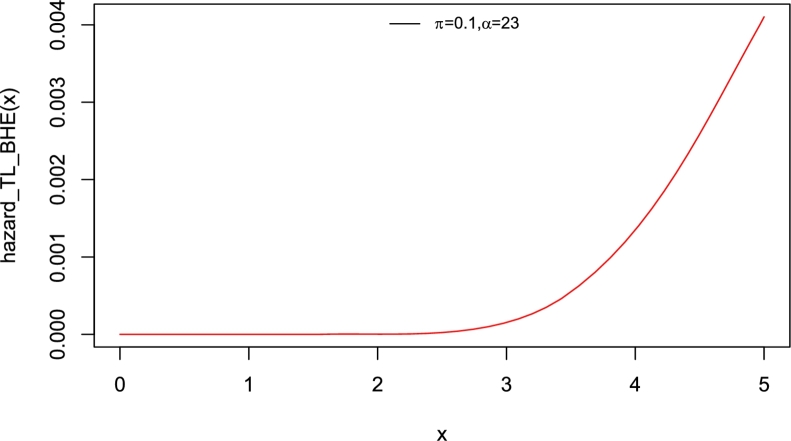
Hazard function of TL-HBE (*π*,*α*).

**Figure 5:**
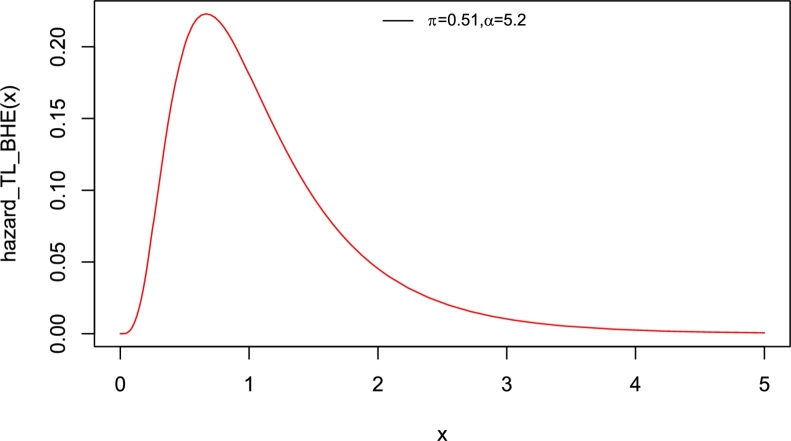
Hazard function of TL-HBE (*π*,*α*).

**Figure 6:**
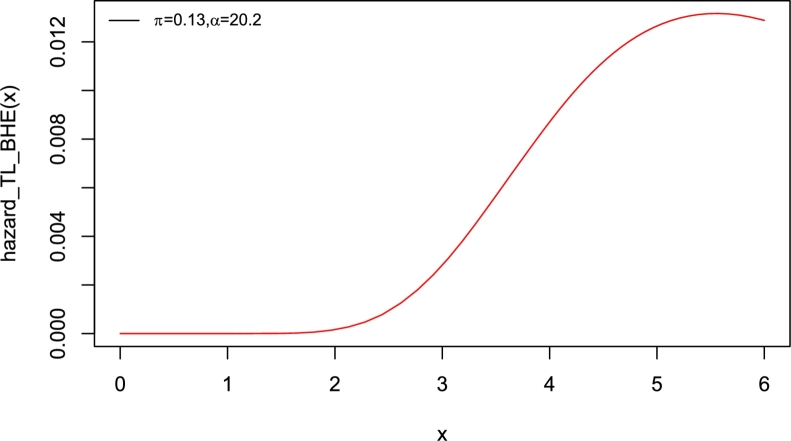
Hazard function of TL-HBE (*π*,*α*).

**Figure 7:**
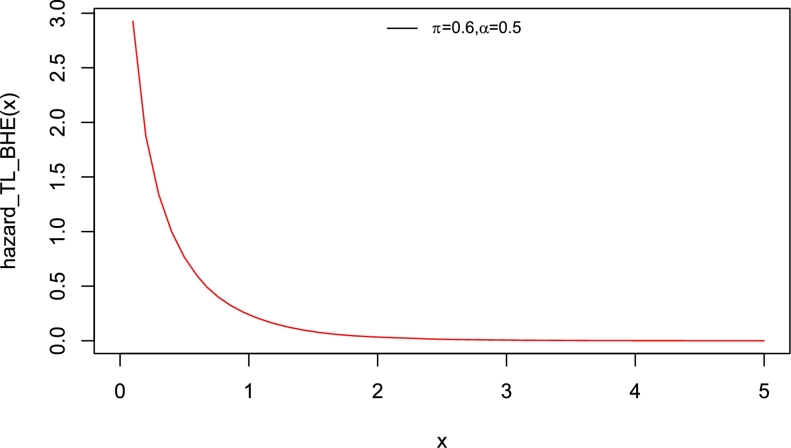
Hazard function of TL-HBE (*π*,*α*).

Interestingly, the baseline distribution has only a scale parameter with right-skewed density and a decreasing hazard rate function (Yadav et al. [55]). Therefore, the new model which has two parameters can also be extended. The motivation to introduce Topp-Leone Burr Hatke exponential (TL-BHE) model based on its flexibility with varying shapes such as left skewed, approximately symmetrical, exponential, and unimodal densities (see Fig. 1). Moreover, the hazard rate function has increasing, decreasing, and reversed bathtub-shaped making it more useful and an alternative to the baseline distribution (see Figure 2, Figure 3, Figure 4, Figure 5, Figure 6, Figure 7).

## 3 Mixture representation and mathematical properties of the model

### 3.1 Mixture representation

The linear representation of the pdf and cdf of the TL-BHE distribution which is useful for the derivation of some properties of the distribution are presented in this section. Firstly, the expansion of the cdf of the TL-BHE distribution is defined in equation (5). using the generalized binomial expansion given by equation (9)

(9) $${\left(1-\zeta \right)}^{\tau }=\sum_{i=0}^{\infty }{\left(-1\right)}^{i}\left(\begin{array}{c} \tau \\ i \end{array}\right)\zeta^{i},|\zeta |<0.$$

where $\zeta =\frac{e^{-\pi x}}{1+\pi x}$ in equation (5).

(10) $$F(x)=\sum_{i=0}^{\infty }{\left(-1\right)}^{i}\left(\begin{array}{c} \alpha \\ i \end{array}\right){\left(\frac{e^{-\pi x}}{1+\pi x}\right)}^{2i}$$

Applying Taylor series expansion $\frac{1}{{\left(1+z\right)}^{j}}=\sum_{j=0}^{\infty }{\left(-1\right)}^{i}\left(\begin{array}{c} j+i-1\\ i \end{array}\right)z^{i}$ to equation (5), equation (10) translates to equation (11)

(11) $$F(x)=\sum_{i,j=0}^{\infty }{\left(-1\right)}^{i+j}\left(\begin{array}{c} \alpha \\ i \end{array}\right)\left(\begin{array}{c} 2i+j-1\\ j \end{array}\right){\left(\pi x\right)}^{j}e^{-2\pi ix}$$

Similarly, the probability density function in (6) can be expressed in linear form as equation (12).

$$f(x)=2\alpha \sum_{i=0}^{\infty }\sum_{j=0}^{\infty }{\left(-1\right)}^{i+j}\left(\begin{array}{c} \alpha -1\\ i \end{array}\right)\left(\begin{array}{c} 2(i+1)+j\\ j \end{array}\right)\left[2\pi^{j+1}x^{j}e^{-2\pi (i+1)x}+\pi^{j+2}x^{j+1}e^{-2\pi (i+1)x}\right]$$

(12) $$=2\alpha \sum_{i=0}^{\infty }\sum_{j=0}^{\infty }Q_{i,j}\left[2\pi^{j+1}x^{j}e^{-2\pi (i+1)x}+\pi^{j+2}x^{j+1}e^{-2\pi (i+1)x}\right]$$

where $Q_{i,j}=2\alpha \sum_{i,j=0}^{\infty }{\left(-1\right)}^{i+j}\left(\begin{array}{c} \alpha -1\\ i \end{array}\right)\left(\begin{array}{c} 2(i+1)+j\\ j \end{array}\right)$.

### 3.2 Mathematical properties

In this section, some mathematical properties of the TL-BHE distribution are derived.

#### 3.2.1 Moments and moment generating functions

Suppose X is a random variable from TL-BHE distribution with probability density function presented in (6), then the $r^{th}$ moments are derived as follows

$$\mu_{r}^{\prime }=E(X^{r})=\int_{0}^{\infty }x^{r}f(x)dx$$

Using the expression in (12), then

(13) $$\mu_{r}^{\prime }=2\alpha \sum_{i=0}^{\infty }\sum_{j=0}^{\infty }Q_{i,j}\int_{0}^{\infty }\left[2\pi^{j+1}x^{j+1}e^{-2\pi (i+1)x}+\pi^{j+2}x^{j+1}e^{-2\pi (i+1)x}\right]dx$$

$$=2\alpha \sum_{i=0}^{\infty }\sum_{j=0}^{\infty }Q_{i,j}2\pi^{j+1}\int_{0}^{\infty }x^{j+r}e^{-2\pi (i+1)x}dx+\pi^{j+2}\int_{0}^{\infty }x^{j+r+1}e^{-2\pi (i+1)x}dx$$

Let u=2π(i+1)x, then if x=0→u=0;x=∞→u=∞, and if $x=\frac{u}{2\pi (i+1)}$, $dx=\frac{du}{2\pi (i+1)}$

(14) $$\mu_{r}^{\prime }=2\alpha \sum_{i=0}^{\infty }\sum_{j=0}^{\infty }Q_{i,j}\left\{\frac{2\pi^{j+1}}{{\left(2\pi (i+1)\right)}^{j+r+1}}\int_{0}^{\infty }u^{j+r+1}e^{-u}du+\frac{\pi^{j+2}}{{\left(2\pi (i+1)\right)}^{j+r+2}}\int_{0}^{\infty }u^{j+r+2}e^{-u}du\right\}$$

Using the well known properties of gamma function defined as $\Gamma (n)=\int_{0}^{\infty }u^{n-1}e^{-u}du$. Let n−1=j+r in (14), then n=j+r+1, for any positive integer *r*. We obtain the moment as

(15) $$\mu_{r}^{\prime }=2\alpha \sum_{i=0}^{\infty }\sum_{j=0}^{\infty }\frac{Q_{i,j}}{2^{j+r}\pi^{r}{\left(i+1\right)}^{j+r+1}}\left[\Gamma (j+r+2)+\frac{\Gamma (j+r+3)}{4(i+1)}\right]$$

For r=1, one obtains the mean of TL-BHE distribution. The $r^{th}$ central moment of TL-BHE distribution

$$\mu_{r}=E{\left(x-\mu \right)}^{r}=\sum_{h=0}^{\infty }{\left(-1\right)}^{h}\left(\begin{array}{c} r\\ h \end{array}\right)\mu_{1}^{\prime }\mu_{r-h}^{\prime }$$

Given a TL-BHE random variable *X*, the moment generating function is obtained as in equation (16).

$$M_{x}(t)=E(e^{tx})=2\alpha \pi \sum_{i=0}^{\infty }\sum_{j=0}^{\infty }Q_{i,j}\left[2\pi^{j+1}\int_{0}^{\infty }x^{j+1}e^{-\left[2\pi (i+1)-t\right]x}dx+\pi^{j+2}\int_{0}^{\infty }x^{j+2}e^{-\left[2\pi (i+1)-t\right]x}dx\right]$$

(16) $$=2\alpha \sum_{i=0}^{\infty }\sum_{j=0}^{\infty }\frac{(j+1)j!\pi^{j+1}Q_{i,j}}{{\left[2\pi (i+1)-t\right]}^{j+1}}\left[1+\frac{\pi (j+2)}{2\pi (i+1)-t}\right]$$

Besides, the characteristic function of the TL-BHE distribution is derived similarly as in equations (16). It given in equation (17)

(17) $$\phi_{X}(t)=2\alpha \sum_{i=0}^{\infty }\sum_{j=0}^{\infty }\frac{(j+1)j!\pi^{j+1}Q_{i,j}}{{\left[2\pi (i+1)-it\right]}^{j+1}}\left[1+\frac{\pi (j+2)}{2\pi (i+1)-it}\right]$$

### 3.3 Entropy

Entropy measure plays a principal part in studying the amount of variation and randomness in a system. In this study, the Renyi entropy (Rényi [41]) shall be employed to determine the amount of randomness in the TL−BHE random variable. Renyi entropy is defined in particular by equation (18)

(18) $$I_{R}(\nu )=\frac{1}{1-\nu }log\int_{0}^{\infty }f_{TL-BHE}^{\nu }(x;\pi ,\alpha )dx,\nu >0,\nu \neq 1$$

inserting (6) into (18), one obtains the Renyi entropy of the TL-BHE model as in equation (19)

(19) $$=\frac{1}{1-\nu }\log\left\{{\left(2\alpha \pi \right)}^{v}\sum_{i,j=0}^{\infty }{\left(-1\right)}^{i+j}\pi^{j}\left(\begin{array}{c} \alpha v-v\\ i \end{array}\right)\left(\begin{array}{c} 2i+j-1\\ j \end{array}\right)\int_{0}^{\infty }x^{j}e^{-2\pi vx}dx\right\}=\frac{1}{1-\nu }\log\left\{{\left(2\alpha \pi \right)}^{v}\sum_{i,j=0}^{\infty }{\left(-1\right)}^{i+j}\pi^{j}\left(\begin{array}{c} \alpha v-v\\ i \end{array}\right)\left(\begin{array}{c} 2i+j-1\\ j \end{array}\right)\frac{\Gamma (j+1)}{{\left(2\pi v\right)}^{j+1}}\right\}$$

### 3.4 Stress strength reliability analysis

Stress strength analysis plays a vital role in reliability engineering as a deciding factor in the failure of components under stress. This stress strength (SS) model can be applied in designing mechanical components in engineering, determining the performance of two biomedical components, and quality control. During the operation of a system certain factors like the operating environment, operators, and external shocks which are regarded as stresses influence the reliability of systems. Interestingly, extensive literature focusing on the study of the Stress Strength model estimation under different scenarios exists. The usefulness and applications of SS can be seen in Bai et al. [10], Jha et al. [27], Shawky and Khan [46], Salah Youssef Temraz [44], Nadar and Erçelik [34], and others. Here, we shall consider single-component and multi-component stress strength.

Single Components Stress-Strength Reliability. Here consideration is given to a situation where a unit operates if the strength of the unit is in excess of the stress. Consider a single-component stress strength model reliability based on two independent random variables *V* and *W*, where *V* represents the “strength” and *W* represents the “stress”. Suppose that *V* and *W* have the $TL-BHE(\pi_{1},\alpha )$ and $TL-BHE(\pi_{2},\alpha )$ distributions with same shape parameter *α* and different scale parameters $\pi_{1}$ and $\pi_{2}$, respectively, then the probability the strength of a system exceeds its stress is given as

$$R_{\left(TL-BHE\right)}=P(W<V)=\int_{0}^{\infty }g_{V}(x)G_{W}(x)dx$$

where $g_{v}(.)$ and $G_{W}(.)$ is the pdf of V and cdf of W respectively

$$=\int_{0}^{\infty }2\alpha \pi_{1}e^{-2\pi_{1}x}\frac{2+\pi_{1}x}{{\left(1+\pi_{1}x\right)}^{3}}{\left[1-{\left(\frac{e^{-\pi_{1}x}}{1+\pi_{1}x}\right)}^{2}\right]}^{\alpha -1}{\left[1-{\left(\frac{e^{-\pi_{2}x}}{1+\pi_{2}x}\right)}^{2}\right]}^{\alpha }dx$$

Using the expansion ${\left(1-z\right)}^{n}=\sum_{i=0}^{\infty }\left(\begin{array}{c} n\\ i \end{array}\right)z^{i}$, leads to equation (20)

$$R_{\left(TL-BHE\right)}=2\alpha \pi_{1}\sum_{i,j,k,l=0}^{\infty }{\left(-1\right)}^{i+j+k+l}\left(\begin{array}{c} \alpha -1\\ i \end{array}\right)\left(\begin{array}{c} \alpha \\ j \end{array}\right)\left(\begin{array}{c} 2(2+i)+k\\ k \end{array}\right)\left(\begin{array}{c} 2i+l-1\\ l \end{array}\right)\pi_{1}^{j}\pi_{2}^{l}\int_{0}^{\infty }x^{j+l}(2+\pi_{1}x)e^{2(i+1)(\pi_{1}+\pi_{2})x}dx$$

(20) $$=2\alpha \pi_{1}\sum_{i,j,k,l=0}^{\infty }{\left(-1\right)}^{i+j+k+l}\left(\begin{array}{c} \alpha -1\\ i \end{array}\right)\left(\begin{array}{c} \alpha \\ j \end{array}\right)\left(\begin{array}{c} 2(2+i)+k\\ k \end{array}\right)\left(\begin{array}{c} 2i+l-1\\ l \end{array}\right)\pi_{1}^{j}\pi_{2}^{l}\left(2\int_{0}^{\infty }x^{j+l}e^{2(i+1)(\pi_{1}+\pi_{2})x}dx+\pi_{1}\int_{0}^{\infty }x^{j+l+1}e^{2(i+1)(\pi_{1}+\pi_{2})x}dx\right)$$

Applying the properties of gamma function, let $\left(u=2(i+1)(\pi_{1}+\pi_{2})x\right)$, if (x=0,u=0), and if (x=∞,u=∞); $\left(du=2(i+1)(\pi_{1}+\pi_{2})dx\right)$, $∴\;\left(dx=\frac{du}{2(i+1)(\pi_{1}+\pi_{2})}\right)$ and $\left(x=\frac{u}{2(i+1)(\pi_{1}+\pi_{2})}\right)$, then equation (21)

(21) $$R_{\left(TL-BHE\right)}=2\alpha \sum_{i,j,k,l=0}^{\infty }\phi_{i,j,k,l}\pi_{1}^{j+1}\pi_{1}^{l}\left\{\frac{\Gamma (j+l+1)}{2^{j+l}{\left((i+1)(\pi_{1}+\pi_{2})\right)}^{j+l+1}}+\frac{\pi_{1}\Gamma (j+l+2)}{{\left(2(i+1)(\pi_{1}+\pi_{2})\right)}^{j+l+2}}\right\}$$

where $\phi_{i,j,k,l}=2\alpha \pi_{1}\sum_{i,j,k,l=0}^{\infty }{\left(-1\right)}^{i+j+k+l}\left(\begin{array}{c} \alpha -1\\ i \end{array}\right)\left(\begin{array}{c} \alpha \\ j \end{array}\right)\left(\begin{array}{c} 2(2+i)+k\\ k \end{array}\right)\left(\begin{array}{c} 2i+l-1\\ l \end{array}\right)\pi_{1}^{j}\pi_{2}^{l}$

Suppose a multi-component system with k components having random strength of $X_{1},X_{2},...,X_{k}$ which are independent and identically distributed random variables—considering each component undergoing random stress. The system's activity will continue provided the strength of at least s (1 ≤ s ≤ k) components out of k components surmounts its stress. A multi-component stress strength (MSS) reliability Model is presented in equation (22)

(22) $$Q_{s,k}=\sum_{i=s}^{k}\left(\begin{array}{c} k\\ i \end{array}\right)\int_{-\infty }^{\infty }{\left[1-F_{W}(x)\right]}^{i}{\left[F_{W}(x)\right]}^{k-1}dF_{V}(x)$$

where $F_{W}(.)$ is the common continuous distribution function of W ($X_{1},X_{2},...,X_{k}$) and V is the common stress with distribution function $F_{V}(.)$ which are independent samples and taken from $TL-BHE(\pi_{1},\alpha )$ and $TL-BHE(\pi_{2},\alpha )$ distributions respectively.

Applying equations (5) and (6), we have that the Multi-component stress strength for TL-BHE is

$$Q_{s,k}(TL-BHE)=2\alpha \pi_{2}\sum_{i=s}^{k}\left(\begin{array}{c} k\\ i \end{array}\right)\int_{0}^{\infty }{\left[1-{\left[1-{\left(\frac{e^{-\pi_{1}x}}{1+\pi_{1}x}\right)}^{2}\right]}^{\alpha }\right]}^{i}{\left[1-{\left(\frac{e^{-\pi_{1}x}}{1+\pi_{1}x}\right)}^{2}\right]}^{k-i}\;e^{-2\pi_{2}x}\frac{2+\pi_{2}x}{{\left(1+\pi_{2}x\right)}^{3}}{\left[1-{\left(\frac{e^{-\pi_{2}x}}{1+\pi_{2}x}\right)}^{2}\right]}^{\alpha -1}$$

Suppose the generalized binomial expansion is considered as ${\left(1-z\right)}^{k}=\sum_{i}^{g=0}\left(\begin{array}{c} k\\ g \end{array}\right)z^{g},|z|<0$ see (Ahmed et al. [4]) then,

$$Q_{s,k}(TL-BHE)=2\alpha \pi_{2}\sum_{i=s}^{k}\sum_{g=0}^{\infty }\sum_{h=0}^{\infty }\sum_{p=0}^{\infty }\sum_{q=0}^{\infty }\sum_{r=0}^{\infty }{\left(-1\right)}^{g+h+p+q+r}\left(\begin{array}{c} k\\ i \end{array}\right)\left(\begin{array}{c} i\\ g \end{array}\right)\left(\begin{array}{c} \alpha g+k-i\\ h \end{array}\right)\left(\begin{array}{c} \alpha -1\\ p \end{array}\right)\left(\begin{array}{c} 3+2p\\ q \end{array}\right)\left(\begin{array}{c} 2h\\ r \end{array}\right){\pi_{1}}^{r}{\pi_{2}}^{q+1}\int_{0}^{\infty }2x^{q+r}e^{-2(\pi_{2}+\pi_{2}p+\pi_{1}h)x}dx+\int_{0}^{\infty }\pi^{2}x^{q+r+1}e^{-2(\pi_{2}+\pi_{2}p+\pi_{1}h)x}dx$$

Let $(u=2(\pi_{2}+\pi_{2}p+\pi_{h})x),ifx=0,u=0;ifx=\infty ,u=\infty ;du=2(\pi_{2}+\pi_{2}p+\pi_{1}h)dx;x=\frac{u}{2(\pi_{2}+\pi_{2}p+\pi_{1}h)}$

Given that $\left(\Gamma n=\int_{0}^{\infty }x^{n-1}e^{-x}dx\right)$, then equation (23) easily follows

(23) $$Q_{s,k}(\text{TL-BHE})=2\alpha \sum_{i=s}^{k}\sum_{g=0}^{\infty }\sum_{h=0}^{\infty }\sum_{p=0}^{\infty }\sum_{q=0}^{\infty }\sum_{r=0}^{\infty }\phi_{i.g,h,p,q,r}\;\left[\frac{\Gamma (q+r+1)}{2^{q+r}{\left(\pi_{2}+\pi_{2}p+\pi_{1}h\right)}^{q+r+1}}+\frac{\pi^{2}\Gamma (q+r+2)}{{\left(2(\pi_{2}+\pi_{2}p+\pi_{1}h)\right)}^{q+r+2}}\right]$$

where $\phi_{i,g,h,p,q,r}={\left(-1\right)}^{g+h+p+q+r}\left(\begin{array}{c} k\\ i \end{array}\right)\left(\begin{array}{c} i\\ g \end{array}\right)\left(\begin{array}{c} \alpha g+k-i\\ h \end{array}\right)\left(\begin{array}{c} \alpha -1\\ p \end{array}\right)\left(\begin{array}{c} 3+2p\\ q \end{array}\right)\left(\begin{array}{c} 2h\\ r \end{array}\right)$.

### 3.5 Order statistics

Let $X_{1},X_{2},...,X_{n}$ be random variables of sizes *n*, each following the TL-BHE distribution, and $x_{\left(1:n\right)},x_{\left(2:n\right)},...,x_{\left(n:n\right)}$ be the analogous order statistics, the $s^{th}$ order statistics has a pdf which is generally expressed in equation (24)

(24) $$f_{s:n}(x)=\frac{1}{B(s,n+s-1)}F^{s-1}(x){\left(1-F(x)\right)}^{n-s}f(x);\;s=1,2,\cdots ,n.$$

Using (5) and (6) in (24), the $s^{th}$ order statistics for the TL-BHE distribution has a pdf obtained as in equation (25)

(25) $$\frac{\alpha }{B(s,n+s-1)}\sum_{i=0}^{n-s}\sum_{j,k,l}^{\infty }\frac{{\left(-1\right)}^{i+j+k+l}}{l!}\left(\begin{array}{c} \alpha (s+i)-1\\ j \end{array}\right)\left(\begin{array}{c} 3+2i\\ k \end{array}\right){\left(j+1\right)}^{l}[2x^{k+l}+\pi x^{k+l+1}]$$

It follows that when we set s=1 and s=n in (25) we obtain the minimum and maximum order statistics for the TL-BHE distribution respectively.

## 4 Non-Bayesian estimation

Here, seven non-bayesian estimation procedures are discussed.

### 4.1 The ML estimation

Let $x_{1},x_{2},...,x_{n}$ be a random sample from the TL−BHE distribution, the likelihood function is the joint probability density of the observed data $x_{i}$ given the parameters *π* and *α*. It can be expressed as in equation (26)

(26) $$L=\prod_{i=1}^{n}f(x_{i};\pi ,\alpha )=\prod_{i=1}^{n}\left[2\alpha \pi e^{-2\pi x_{i}}\frac{2+\pi x_{i}}{{\left(1+\pi x_{i}\right)}^{3}}{\left(1-{\left(\frac{e^{-\pi x_{i}}}{1+\pi x_{i}}\right)}^{2}\right)}^{\alpha -1}\right]$$

The corresponding log-likelihood function is in equation (27)

(27) $$\ln L=n\ln(2)+n\ln(\alpha )+n\ln(\pi )-2\pi \sum_{i=0}^{n}x_{i}+\sum_{i=0}^{n}\ln(2+\pi x_{i})-3\sum_{i=0}^{n}\ln(1+\pi x_{i})+(\alpha -1)\sum_{i=0}^{n}\ln\left[1-{\left(\frac{e^{-\pi x_{i}}}{1+\pi x_{i}}\right)}^{2}\right]$$

Therefore, the maximum likelihood estimate of *α* and *π* can be derived by solving the minimized nonlinear equations in (28) and (29)

(28) $$\frac{\partial lnL}{\partial \alpha }=\frac{n}{\alpha }+\sum_{i=0}^{n}ln\left(1-{\left(\frac{e^{-\pi x_{i}}}{1+\pi x_{i}}\right)}^{2}\right)=0$$

(29) $$\frac{\partial lnL}{\partial \pi }=\frac{n}{\pi }-2\sum_{i=0}^{n}x_{i}+\sum_{i=0}^{n}\frac{x_{i}}{\left(2+\pi x_{i}\right)}-3\sum_{i=0}^{n}\frac{x_{i}}{\left(1+\pi x_{i}\right)}+2(\alpha -1)\sum_{i=0}^{n}\frac{(2+\pi x_{i})x_{i}e^{-2\pi x_{i}}}{{\left(1+\pi x_{i}\right)}^{3}}=0$$

Since equations (28) and (29) cannot be solved analytically, numerical iteration approaches like the Newton-Raphson algorithm in the **“MaxLik”** R-package (Team [50]) are utilized to derive the estimates.

### 4.2 The MPS estimation

Here, consideration is given to data presented in increasing order of magnitude, the TL-BHE greatest product spacing is presented in equation (30)

(30) $$G_{i:m:n}(\pi ,\alpha |x_{i:n})={\left(\prod_{i=1}^{n+1}D_{i}(x_{i:n},\pi ,\alpha )\right)}^{\frac{1}{n+1}}$$

where $D_{i}(x_{i:n},\pi ,\alpha )=F(x_{i:n};\pi ,\alpha )-F(x_{i-1};\pi ,\alpha )\;i=1,2,\cdots ,n$, provided the sample is random and ordered; that is $x_{1:n},x_{2:n},\cdots ,x_{n:n}$.

If we increase the function represented in equation (31)

(31) $$S(\pi ,\alpha )=\frac{1}{n+1}\sum_{i=1}^{n+1}\ln D_{i}(\pi ,\alpha )$$

and solve for the first derivative with respect to *π* and *α* the parameter estimates are obtained. That is $\frac{\partial S(\xi )}{\partial \pi }=0$ and $\frac{\partial S(\xi )}{\partial \alpha }=0$, where ξ=(π,α).

### 4.3 The LS estimation

Consider the order statistics of a random sample $x_{1:n},x_{2:n},\cdots ,x_{n:n}$ from the TL-BHE distribution, the LSEs of the TL-BHE parameters (π,α) are obtained by minimizing the function L(π,α) as in equation (32)

(32) $$L(\pi ,\alpha )=\underset{\left(\pi ,\alpha \right)}{\arg\min}\sum_{i=1}^{n}{\left[F(x_{i:n}|\pi ,\alpha )-\frac{i}{n+1}\right]}^{2}.$$

See Swain et al. [49] for more details. To get the least squares estimates for the parameters, we solve these system of non-linear equations presented in equations (33) and (34)

(33) $$\sum_{i=1}^{n}{\left[F(x_{i:n}|\pi ,\alpha )-\frac{i}{n+1}\right]}^{2}\Delta_{1}(x_{i:n}|\pi ,\alpha )=0$$

(34) $$\sum_{i=1}^{n}{\left[F(x_{i:n}|\pi ,\alpha )-\frac{i}{n+1}\right]}^{2}\Delta_{2}(x_{i:n}|\pi ,\alpha )=0$$

where

(35) $$\Delta_{2}(x_{i:n}|\pi ,\alpha )=\frac{2\alpha x_{i:n}(2+\pi x_{i:n})e^{-2\pi x_{i:n}}}{{\left(1+\pi x_{i:n}\right)}^{3}}{\left[1-{\left(\frac{e^{-\pi x_{i:n}}}{1+\pi x_{i:n}}\right)}^{2}\right]}^{\alpha -1},$$

and

(36) $$\Delta_{1}(x_{i:n}|\pi ,\alpha )={\left[1-{\left(\frac{e^{-\pi x_{i:n}}}{1+\pi x_{i:n}}\right)}^{2}\right]}^{\alpha }\ln\left[1-{\left(\frac{e^{-\pi x_{i:n}}}{1+\pi x}\right)}^{2}\right].$$

Equations (35) and (36) are obtained from the partial differentiation of equation (5) with respect to *π* and *α*.

### 4.4 The WLS estimation

If the function W(π,α) is minimized the estimates ${\overset{ˆ}{\pi }}_{WLSE}$ and ${\overset{ˆ}{\alpha }}_{WLSE}$ of the proposed TL-BHE parameters *π* and *α* are obtained as given in equation (37)

(37) $$W(\pi ,\alpha )=\underset{\left(\pi ,\alpha \right)}{\arg\min}\sum_{i=1}^{n}\frac{{\left(n+1\right)}^{2}(n+2)}{i(n-i+1)}{\left[F(x_{i:n}|\pi ,\alpha )-\frac{i}{n+1}\right]}^{2}.$$

This system of non-linear equations in (38) and (39) solved iteratively give the weighted least squares estimates

(38) $$\sum_{i=1}^{n}\frac{{\left(n+1\right)}^{2}(n+2)}{i(n-i+1)}[F(x_{i:n}|\pi ,\alpha )-\frac{i}{n+1}]\Delta_{1}(y_{i:n}|\pi ,\alpha )=0$$

(39) $$\sum_{i=1}^{n}\frac{{\left(n+1\right)}^{2}(n+2)}{i(n-i+1)}[F(x_{i:n}|\pi ,\alpha )-\frac{i}{n+1}]\Delta_{2}(x_{i:n}|\pi ,\alpha )=0,$$

$\Delta_{1}(x|\pi ,\alpha )$ and $\Delta_{2}(x|\pi ,\alpha )$ are respectively defined in (35) and (36).

### 4.5 The CVM estimation

We get the estimates of ${\overset{ˆ}{\pi }}_{CVM}$, and ${\overset{ˆ}{\alpha }}_{CVM}$ for the TL-BHE parameters *π*, and *α* when we minimize equation (40)

(40) $$C(\pi ,\alpha )=\underset{\left(\pi ,\alpha \right)}{\arg\min}\{\frac{1}{12n}+\sum_{i=1}^{n}{\left[F(x_{i:n}|\pi ,\alpha )-\frac{2i-1}{2n}\right]}^{2}\},$$

we again solve the non-linear system of equations in (41)

(41) $$\begin{array}{cc} \sum_{i=1}^{n}(F(x_{i:n}|\pi ,\alpha )-\frac{2i-1}{2n})\Delta_{1}(x_{i:n}|\pi ,\alpha ) & =0\\ \sum_{i=1}^{n}(F(x_{i:n}|\pi ,\alpha )-\frac{2i-1}{2n})\Delta_{2}(x_{i:n}|\pi ,\alpha ) & =0, \end{array}\}$$

for $\Delta_{1}(x|\pi ,\alpha )$ and $\Delta_{2}(x|\pi ,\alpha )$ are respectively defined in (35) and (36).

### 4.6 The AD estimation

The estimates ${\overset{ˆ}{\pi }}_{AD}$, and ${\overset{ˆ}{\alpha }}_{ADE}$ of the TL-BHE parameters *π* and *α* are obtained by minimizing the function A(π,α) as in equation (42)

(42) $$A(\pi ,\alpha )=\underset{\left(\pi ,\alpha \right)}{\arg\min}\sum_{i=1}^{n}(2i-1)\{\ln F(x_{i:n}|\pi ,\alpha )+\ln[1-F(x_{n+1-i:n}|\pi ,\alpha )]\}.$$

Solving the following systems of non-linear equations (43) produces estimates

(43) $$\begin{array}{cc} \sum_{i=1}^{n}(2i-1)[\frac{\Delta_{1}(x_{i:n}|\pi ,\alpha )}{F(x_{i:n}|\pi ,\alpha )}-\frac{\Delta_{1}(x_{n+1-i:n}|\pi ,\alpha )}{1-F(x_{n+1-i:n}|\pi ,\alpha )}] & =0\\ \sum_{i=1}^{n}(2i-1)[\frac{\Delta_{2}(x_{i:n}|\pi ,\alpha )}{F(y_{i:n}|\pi ,\alpha )}-\frac{\Delta_{2}(y_{n+1-i:n}|\pi ,\alpha )}{1-F(x_{n+1-i:n}|\pi ,\alpha )}] & =0, \end{array}\}$$

where $\Delta_{1}(x|\pi ,\alpha )$ and $\Delta_{2}(x|\pi ,\alpha )$ is as defined in (35) and (36) respectively.

### 4.7 The RTAD estimation

In similar fashion we did in AD method, R(π,α) is minimized to get ${\overset{ˆ}{\pi }}_{RTADE}$ and ${\overset{ˆ}{\alpha }}_{RTAD}$ so that from equation (44)

(44) $$R(\pi ,\alpha )=\underset{\left(\pi ,\alpha \right)}{\arg\min}\{\frac{n}{2}-2\sum_{i=1}^{n}F(x_{i:n}|\pi ,\alpha )-\frac{1}{n}\sum_{i=1}^{n}(2i-1)\ln[1-F(x_{n+1-i:n}|\pi ,\alpha )]\},$$

we solve equation (45)

(45) $$\begin{array}{cc} -2\sum_{i=1}^{n}\frac{\Delta_{1}(x_{i:n}|\pi ,\alpha )}{F(x_{i:n}|\pi ,\alpha )}+\frac{1}{n}\sum_{i=1}^{n}(2i-1)[\frac{\Delta_{1}(x_{n+1-i:n}|\pi ,\alpha )}{1-F(x_{n+1-i:n}|\pi ,\alpha )}] & =0\\ -2\sum_{i=1}^{n}\frac{\Delta_{2}(x_{i:n}|\pi ,\alpha )}{F(x_{i:n}|\pi ,\alpha )}+\frac{1}{n}\sum_{i=1}^{n}(2i-1)[\frac{\Delta_{2}(x_{n+1-i:n}|\pi ,\alpha )}{1-F(y_{n+1-i:n}|\pi ,\alpha )}] & =0, \end{array}\}$$

for $\Delta_{1}(x|\pi ,\alpha )$ and $\Delta_{2}(x|\pi ,\alpha )$ are respectively defined in (35) and (36), we obtain the RTAD estimates of the parameters.

## 5 Bayesian estimation

Handling conditional probability has become seamless due to the availability of prior information about the data. Comparing the likelihood of two events occurring in the past can hence be used to ascertain the future likelihood. Using the informative prior, the Bayes estimates of the parameters (π,α) are obtained easily. The most popular informative priors is the gamma prior and we deploy it here for the *π* and *α* now variables with PDFs in the prior distribution of TL-BHE. In Bayesian analysis, the choice of prior distribution is crucial as it reflects prior beliefs about the parameters before observing the data. An informative prior is particularly useful when there is existing knowledge about the parameter, and it can significantly influence the posterior distribution. The Gamma distribution is commonly used as an informative prior, especially when modeling parameters that are positive and continuous, such as rates or scale parameters. The Gamma distribution is defined as:

$$\text{Gamma}(\alpha ,\beta )=\frac{\beta^{\alpha }}{\Gamma (\alpha )}x^{\alpha -1}e^{-\beta x},\;x>0,\alpha >0,\beta >0$$

where *α* (shape) and *β* (rate) are hyperparameters that control the shape and scale of the distribution.

**Justification for Using a Gamma Prior:**

1. The Gamma prior is chosen when there is prior knowledge or expert opinion about the parameter being modeled. For instance, in modeling rates (e.g., failure rates, hazard rates), where historical data or domain expertise suggests a specific range or behavior for the parameter, the Gamma prior allows incorporating this information effectively.
2. The Gamma prior is conjugate to several likelihood functions, such as the Poisson, Exponential, and Normal with known variance. This conjugacy simplifies the computation of the posterior distribution, making the Bayesian analysis more tractable.
3. The Gamma distribution is flexible due to its shape and scale parameters. It can represent a wide range of prior beliefs, from highly concentrated (informative) to diffuse (less informative). This flexibility makes it a robust choice for various applications.
4. The parameters of the Gamma distribution have intuitive interpretations. The shape parameter *α* controls the concentration of the distribution, while the rate parameter *β* controls the spread. This makes it easier to encode and communicate prior beliefs.

Hence, the Gamma prior is an appropriate choice for Bayesian analysis when prior information about a positive parameter is available. It allows for the incorporation of this information in a mathematically convenient and interpretable way, while also offering flexibility and conjugacy properties that facilitate the analytical and computational aspects of Bayesian inference. The gamma priors using the parameters as random variables is in equation (46)

(46) $$\pi_{1}(\pi )\propto \pi^{\omega_{1}-1}e^{-t_{1}\pi };\;\pi >0,\;\omega_{1}>0,\;t_{1}>0\;\pi (\alpha )\propto \alpha^{\omega_{2}-1}e^{-t_{2}\alpha };\;\alpha >0,\;\omega_{2}>0,\;t_{2}>0$$

Note that the hyper-parameters $\omega_{s},t_{s},s=1,2$ are such that the prior information about the unknown parameters is rejected. Though the BEs depend on the choice of hyper-parameters $\omega_{s}$ and $t_{s}$ yet, we can use the empirical Bayes technique introduced by Awad and Gharraf [9] to estimate the hyper-parameters. The joint prior for ξ=(π,α) is given by equation (47)

(47) $$\pi (\xi )=\pi_{1}(\pi )\pi_{2}(\alpha )\pi (\xi )\propto \pi^{\omega_{1}-1}\alpha^{\omega_{2}-1}e^{\left\{-t_{1}\pi -t_{2}\alpha \right\}}$$

The associated posterior distribution given observation $x=(x_{1},x_{2},...,x_{n})$ is

$$\pi (\xi |\text{y})=\frac{\pi (\xi )\ell (\xi )}{\int_{\xi }\pi (\xi )\ell (\xi )d\xi }$$

Hence, the posterior density function is obtained as in equation (48)

(48) $$\pi (\xi |\text{x})\propto \pi^{\omega_{1}-1}\alpha^{\omega_{2}-1}e^{\left\{-t_{1}\pi -t_{2}\alpha \right\}}\prod_{i=1}^{n}\left[2\alpha \pi e^{-2\pi x_{i}}\frac{2+\pi x_{i}}{{\left(1+\pi x_{i}\right)}^{3}}{\left(1-{\left(\frac{e^{-\pi x_{i}}}{1+\pi x_{i}}\right)}^{2}\right)}^{\alpha -1}\right]$$

For l(ξ) based on SEL, the BE is

(49) $${\overset{ˆ}{\xi }}_{BE_{SEL}}=E[l(\omega )|\text{x}]=\int_{\xi }l(\xi )\pi (\xi |x)d\xi$$

One challenge with the use of SEL is in handling over- and under-estimation with equal weights. Both sides of the divide may have important considerations before right choices are made. The linear exponential loss function (LINEX) is an alternative measure given as

$$(l(\xi ),\overset{ˆ}{l}(\xi ))=e^{\left\{\overset{ˆ}{l}(\xi )-l(\xi )\right\}}-\tau (\overset{ˆ}{l}(\xi )-l(\xi ))-1$$

with shape parameter τ≠0, and τ>1 means that overestimation is weightier than an underestimation. The reverse is the case for τ>0. When τ→0, the recursive iteration achieves the SEL function, see Varian [53] and Doostparast et al. [14]. The Bayes estimates l(ξ) based on this loss function is

(50) $$\xi_{BE_{LINEX}}=E[e^{\left\{-\tau (\xi )\right\}}|\text{x}]=-\frac{1}{\tau }\log\left[\int_{\xi }e^{\left\{-\tau (\xi )\right\}}\pi (\xi |x)d\xi \right]$$

Next, consider the generalized entropy loss (GEL) function as proposed by Calabria and Pulcini [12] expressed as

$$(l(\xi ),\overset{ˆ}{l}(\xi ))={\left(\frac{\overset{ˆ}{l}(\xi )}{l(\xi )}\right)}^{\tau }-\tau \log\left(\frac{\overset{ˆ}{l}(\xi )}{l(\xi )}\right)-1.$$

For τ≠0 asymmetry occurs. Again, the loss function considers overestimation to be weightier than underestimation when ν>0 and the reverse is the case for τ<0. The Bayes estimator for l(ξ) based on the GEL is

(51) $$\xi_{BE_{GEL}}={\left[E({\left(l(\xi )\right)}^{-\tau }|x)\right]}^{\frac{-1}{\tau }}={\left[\int_{\xi }{\left(l(\xi )\right)}^{-\tau }\pi (\xi |x)d\xi \right]}^{-\frac{1}{\tau }}$$

The Markov chain Monte Carlo (MCMC) method for generating posterior samples for appropriate BEs is then used when equations (49), (50) and (51) lack tractability in computation. The Markov Chain Monte Carlo (MCMC) algorithm, specifically the Metropolis-Hastings algorithm is provided next.

### 5.1 Metropolis-Hastings (MH) algorithm

1. **Initialize:** Choose an initial point $x_{0}$ and set t=0.
2. **Repeat:**
  (a) **Proposal:** Generate a candidate point $x^{\prime }$ from a proposal distribution $q(x^{\prime }|x_{t})$.
  (b) **Acceptance Probability:** Compute the acceptance probability
    $$\alpha =\min\left(1,\frac{\pi (x^{\prime })q(x_{t}|x^{\prime })}{\pi (x_{t})q(x^{\prime }|x_{t})}\right),$$
    where π(x) is the target distribution.
  (c) **Acceptance/Rejection:** Generate a uniform random number u∼Uniform(0,1). If u≤α, accept the candidate and set $x_{t+1}=x^{\prime }$; otherwise, reject the candidate and set $x_{t+1}=x_{t}$.
  (d) **Increment:** Set t=t+1.
3. **Output:** After a sufficient number of iterations, the chain $\left\{x_{t}\right\}$ will approximate the target distribution π(x).

Next, the uncertainty with the posterior density is measured based on the parameter vector *ξ*. This is achieved using kernel estimate of the posterior density function as well as MCMC samples. We drop some of the initial samples from the *N* samples generated using the posterior density via burn-in while the Bayes estimates are computed from the remaining samples. Through MH algorithm for the SEL, LINEX which is asymmetric, and GEL also asymmetric, the BE_*s*_ of $\xi^{i}={\left(\pi \right)}^{i},{\left(\alpha \right)}^{i}$ are computed in equations (52), (53) and (54),

(52) $${\overset{ˆ}{\xi }}_{BE_{SEL}}=\frac{1}{M-l_{b}}\sum_{i=l_{b}}^{N}\xi^{\left(i\right)},$$

(53) $${\overset{ˆ}{\xi }}_{BE_{LINEX}}=-\frac{1}{\nu }\log\left[\frac{1}{N-l_{b}}\sum_{i=l_{b}}^{N}e^{\left\{-\nu \xi^{\left(i\right)}\right\}}\right],$$

(54) $${\overset{ˆ}{\xi }}_{BE_{GEL}}={\left[\frac{1}{N-l_{b}}\sum_{i=l_{b}}^{N}{\left(\xi^{\left(i\right)}\right)}^{-\tau }\right]}^{-\frac{1}{\tau }},$$

and $l_{b}$ represents the number of burn-in samples. The essence of burn-in is to give the Markov Chain time to attain the equilibrium distribution point, especially if it has a lousy starting point. This is achieved by discarding the first *n* samples before points are recorded. This prevents over-sampling a region with very low probability under the equilibrium distribution before it settles into the equilibrium distribution due to bad initial guess.

### 5.2 Baye's estimate (BE) credible interval (CI)

We calculate a 100(1−γ)% CI for ξ=(π,α) based on the three loss functions studied are in equation (55)

(55) $${\overset{ˆ}{\xi }}_{BE_{SEL}}\pm Z_{\frac{\gamma }{2}}\sqrt{var\left\{\frac{1}{M-l_{b}}\sum_{i=l_{b}}^{N}\xi^{\left(i\right)}\right\}};\;{\overset{ˆ}{\xi }}_{BE_{LINEX}}\pm Z_{\frac{\gamma }{2}}\sqrt{var\left\{-\frac{1}{\nu }\log\left[\frac{1}{N-l_{b}}\sum_{i=l_{b}}^{N}e^{\left\{-\nu \xi^{\left(i\right)}\right\}}\right]\right\}};{\overset{ˆ}{\xi }}_{BE_{GEL}}\pm Z_{\frac{\gamma }{2}}\sqrt{var\left\{{\left[\frac{1}{N-l_{b}}\sum_{i=l_{b}}^{N}{\left(\xi^{\left(i\right)}\right)}^{-\tau }\right]}^{-\frac{1}{\tau }}\right\}},$$

where $Z_{\frac{\gamma }{2}}$ represents the critical value from the standard normal distribution at the $\frac{\gamma }{2}$ percentile, corresponding to the right-tailed probability.

### 5.3 Simulation studies

Simulation of data for the TL-BHE distribution is carried out in this section to provide justification for the flexibility of the traditional non-Bayesian procedures and this is done by generating 1000 data from the TL-BHE with following initial guesses;

• π=0.70 and α=1.5
• π=0.50 and α=1.5
• π=0.50 and α=2.0
• π=0.70 and α=2.0

at sample size n=50,100,150,200 and the estimates $\overset{ˆ}{\xi }=(\overset{ˆ}{\pi },\overset{ˆ}{\alpha })$, we can obtain the Bias and Root Mean Squared Error (RMSE) respectively as

$$Bias(\overset{ˆ}{\xi })=\frac{1}{N}\sum_{i=1}^{N}({\overset{ˆ}{\xi }}_{i}-\xi ),\;\text{and}\;RMSE(\overset{ˆ}{\xi })=\sqrt{\frac{1}{N}\sum_{i=1}^{N}{\left({\overset{ˆ}{\xi }}_{i}-\xi \right)}^{2}}.$$

Since the non-bayesian estimators do not have analytical solutions, the Newton-Raphson algorithm is deployed to obtain convergence for the estimates. For the Bayesian method, the Metropolis-Hastings algorithm and the MCMC principles are used based on prior knowledge of the data to obtain the Bayes estimates. The hyper-parameters used are based on Gamma distribution priors viz v=−1.5,1.5 under squared error and linear exponential loss functions and τ=−0.5,0.5 under the generalized entropy loss function and we remove 2000 burn-in samples from the entire 10000 samples generated from the posterior density function.

The simulation results presented in Table 1, Table 2, Table 3, Table 4 lead to the following inference.

1. The chosen initial parameters for *α* and *π* provided good performance.
2. The estimates perform well as sample size increases. That is
  (a) As the sample size increases, the estimates become more accurate, converging closer to the true values of the parameters being estimated.
  (b) Larger sample sizes generally lead to estimates with less bias. Bias refers to the difference between the expected value of the estimator and the true value of the parameter. Well-performing estimates show decreasing bias as the sample size increases.
  (c) With a larger sample size, the estimates exhibit less variability, in this sense, repeated samples yield more consistent estimates, reducing the standard error.
  (d) As the sample size increases, the empirical distribution converges to the true distribution, making estimates for functions like the probability density function (PDF) and cumulative distribution function (CDF) more accurate.
  (e) Larger sample sizes lead to narrower confidence intervals, providing greater certainty about the true parameter values.
  (f) Larger sample sizes increase the power of statistical tests, making it easier to detect significant effects or differences when they exist.
  Overall, this suggests that the estimation methods being used are reliable and yield results that increasingly reflect the true characteristics of the population or process being studied as more data become available.
3. The Bias and RMSE for the parameters are relatively small.
4. As the sample size becomes large, the Bias and RMSE reduce. This implies an improved estimation accuracy. For all sample sizes, the Bias is positive.

From Table 5, the BEs based on gamma priors are more predictable than those ML. This is also the case with the HPD credible interval.

**Table 1:** Average bias and RMSE for the TL-BHE for various *n* and (*π* = 0.70,*α* = 1.5).

Method		n=50		n=100		n=150		n=200	
		Bias	RMSE	Bias	RMSE	Bias	RMSE	Bias	RMSE
ML	*π*	0.03716	0.02160	0.01907	0.01032	0.01056	0.00686	0.00978	0.00437
	*α*	0.10984	0.15738	0.05326	0.06189	0.03429	0.04085	0.02758	0.02634

MPS	*π*	0.04340	0.01849	0.02785	0.00961	0.02329	0.00677	0.01734	0.00430
	*α*	0.09339	0.11059	0.06334	0.05182	0.04983	0.03679	0.03935	0.02428

LS	*π*	0.00347	0.02858	0.00343	0.01398	0.00127	0.00896	0.00286	0.00614
	*α*	0.03775	0.20148	0.01911	0.08782	0.00879	0.05514	0.01037	0.03790

WLS	*π*	0.01172	0.02381	0.00877	0.01178	0.00409	0.00753	0.00608	0.00503
	*α*	0.05109	0.16655	0.02939	0.07233	0.01986	0.04556	0.01757	0.03072

CVM	*π*	0.03976	0.03312	0.02128	0.01511	0.01047	0.00936	0.01169	0.00642
	*α*	0.13684	0.25967	0.06594	0.09989	0.03928	0.05987	0.03311	0.04054

AD	*π*	0.01656	0.02264	0.00885	0.01120	0.00324	0.00729	0.00557	0.00493
	*α*	0.06147	0.15573	0.02897	0.06790	0.01767	0.04406	0.01627	0.02982

RTAD	*π*	0.03014	0.02812	0.01366	0.01317	0.00708	0.00872	0.00653	0.00571
	*α*	0.12749	0.26193	0.05423	0.10593	0.03677	0.06735	0.02370	0.04273

BE${}_{SEL}$	*π*	0.24102	0.07965	0.27530	0.08641	0.29046	0.09170	0.29696	0.09360
	*α*	0.66925	0.62057	0.79316	0.71979	0.84657	0.78351	0.86828	0.80619

BE${}_{Linex1}$	*π*	0.24604	0.08247	0.27837	0.08823	0.29267	0.09305	0.29869	0.09466
	*α*	0.71333	0.69895	0.82243	0.77315	0.86835	0.82402	0.88548	0.83840

BE${}_{Linex2}$	*π*	0.23608	0.07693	0.27226	0.08463	0.28825	0.09037	0.29523	0.09254
	*α*	0.62827	0.55367	0.76523	0.67109	0.82553	0.74552	0.85153	0.77553

BE${}_{GEL1}$	*π*	0.23581	0.07703	0.27220	0.08466	0.28824	0.09040	0.29523	0.09256
	*α*	0.65074	0.59270	0.78110	0.69951	0.83767	0.76782	0.86125	0.79357

BE${}_{GEL2}$	*π*	0.22539	0.07201	0.26597	0.08123	0.28379	0.08781	0.29177	0.09050
	*α*	0.61421	0.54030	0.75716	0.66026	0.81994	0.73714	0.84724	0.76875

**Table 2:** Average bias and RMSE for the TL-BHE for various *n* and (*π* = 0.50,*α* = 1.5).

Method		n=50		n=100		n=150		n=200	
		Bias	RMSE	Bias	RMSE	Bias	RMSE	Bias	RMSE
ML	*π*	0.02393	0.01054	0.00920	0.00456	0.00771	0.00339	0.00781	0.00244
	*α*	0.10969	0.14700	0.03497	0.05215	0.03026	0.03574	0.03193	0.02705

MPS	*π*	0.03315	0.00923	0.02408	0.00458	0.01662	0.00334	0.01161	0.00236
	*α*	0.09261	0.10330	0.07953	0.04820	0.05385	0.03295	0.03537	0.02436

LS	*π*	0.00075	0.01389	0.00161	0.00637	0.00065	0.00427	0.00222	0.00350
	*α*	0.02668	0.17663	0.00027	0.07169	0.00764	0.04787	0.01407	0.03997

WLS	*π*	0.00574	0.01184	0.00234	0.00524	0.00339	0.00363	0.00493	0.00287
	*α*	0.04534	0.15066	0.01125	0.05771	0.01560	0.03922	0.02213	0.03188

CVM	*π*	0.02508	0.01595	0.01107	0.00682	0.00908	0.00449	0.00852	0.00365
	*α*	0.12468	0.22710	0.04630	0.08054	0.03812	0.05209	0.03688	0.04288

AD	*π*	0.00812	0.01104	0.00232	0.00500	0.00321	0.00356	0.00453	0.00279
	*α*	0.05299	0.14251	0.01102	0.05470	0.01497	0.03839	0.02075	0.03111

RTAD	*π*	0.01413	0.01287	0.00586	0.00592	0.00496	0.00404	0.00597	0.00324
	*α*	0.10061	0.22676	0.03694	0.08795	0.02800	0.05539	0.03189	0.04615

BE${}_{SEL}$	*π*	0.18007	0.04384	0.20105	0.04595	0.21042	0.04807	0.21452	0.04881
	*α*	0.69361	0.65990	0.80759	0.74463	0.85652	0.80133	0.87663	0.82138

BE${}_{Linex1}$	*π*	0.18268	0.04493	0.20263	0.04663	0.21156	0.04857	0.21541	0.04920
	*α*	0.73886	0.74308	0.83721	0.79955	0.87849	0.84268	0.89395	0.85411

BE${}_{Linex2}$	*π*	0.17750	0.04278	0.19948	0.04528	0.20928	0.04757	0.21364	0.04842
	*α*	0.65159	0.58894	0.77932	0.69450	0.83529	0.76255	0.85978	0.79023

BE${}_{GEL1}$	*π*	0.17632	0.04243	0.19882	0.04504	0.20883	0.04739	0.21329	0.04827
	*α*	0.67484	0.63064	0.79546	0.72386	0.84757	0.78538	0.86958	0.80860

BE${}_{GEL2}$	*π*	0.16883	0.03973	0.19437	0.04324	0.20564	0.04605	0.21081	0.04721
	*α*	0.63780	0.57559	0.77137	0.68368	0.82976	0.75418	0.85554	0.78347

**Table 3:** Average bias and RMSE for the TL-BHE for various *n* and (*π* = 0.50,*α* = 2.0).

Method		n=50		n=100		n=150		n=200	
		Bias	RMSE	Bias	RMSE	Bias	RMSE	Bias	RMSE
ML	*π*	0.02252	0.00985	0.01248	0.00463	0.00948	0.00308	0.00631	0.00205
	*α*	0.16167	0.30508	0.07472	0.12602	0.05646	0.07947	0.03850	0.05415

MPS	*π*	0.03185	0.00879	0.01956	0.00439	0.01379	0.00295	0.01211	0.00204
	*α*	0.13258	0.20883	0.09464	0.10539	0.06637	0.06912	0.05833	0.04995

LS	*π*	0.00246	0.01224	0.00152	0.00619	0.00187	0.00402	0.00104	0.00282
	*α*	0.04923	0.41979	0.02599	0.18302	0.02338	0.11443	0.00354	0.07687

WLS	*π*	0.00415	0.01060	0.00537	0.00513	0.00462	0.00342	0.00249	0.00230
	*α*	0.07449	0.35220	0.04070	0.14509	0.03382	0.09379	0.01969	0.06156

CVM	*π*	0.02195	0.01390	0.01361	0.00667	0.00988	0.00424	0.00491	0.00290
	*α*	0.19402	0.55007	0.09431	0.20999	0.06815	0.12579	0.03635	0.08183

AD	*π*	0.00654	0.00987	0.00543	0.00491	0.00462	0.00332	0.00198	0.00225
	*α*	0.08120	0.30328	0.04018	0.13555	0.03339	0.08993	0.01673	0.05941

RTAD	*π*	0.01547	0.01334	0.00861	0.00603	0.00744	0.00397	0.00399	0.00283
	*α*	0.19065	0.67291	0.07818	0.22464	0.06272	0.14172	0.03763	0.09387

BE${}_{SEL}$	*π*	0.14252	0.02886	0.17248	0.03414	0.18632	0.03781	0.19309	0.03959
	*α*	0.83144	0.98358	1.07113	1.31777	1.18140	1.52887	1.23333	1.62780

BE${}_{Linex1}$	*π*	0.14459	0.02954	0.17377	0.03461	0.18728	0.03819	0.19385	0.03989
	*α*	0.91326	1.16842	1.12954	1.46348	1.22678	1.64820	1.26997	1.72625

BE${}_{Linex2}$	*π*	0.14046	0.02819	0.17119	0.03367	0.18537	0.03745	0.19234	0.03929
	*α*	0.75742	0.83543	1.01654	1.19023	1.13829	1.42052	1.19820	1.53660

BE${}_{GEL1}$	*π*	0.13934	0.02792	0.17057	0.03347	0.18494	0.03729	0.19201	0.03917
	*α*	0.80542	0.93490	1.05335	1.27737	1.16784	1.49546	1.22248	1.60005

BE${}_{GEL2}$	*π*	0.13298	0.02612	0.16674	0.03215	0.18217	0.03626	0.18985	0.03833
	*α*	0.75412	0.84408	1.01808	1.19948	1.14089	1.43029	1.20085	1.54558

**Table 4:** Average bias and RMSE for the TL-BHE for various *n* and (*π* = 0.70,*α* = 2.0).

Method		n=50		n=100		n=150		n=200	
		Bias	RMSE	Bias	RMSE	Bias	RMSE	Bias	RMSE
ML	*π*	0.02730	0.01755	0.01458	0.00785	0.00992	0.00539	0.00683	0.00419
	*α*	0.14122	0.28712	0.07801	0.11652	0.05562	0.07514	0.04672	0.05553

MPS	*π*	0.04866	0.01640	0.03005	0.00778	0.02254	0.00539	0.01897	0.00425
	*α*	0.15014	0.20470	0.09108	0.09750	0.06712	0.06577	0.05090	0.04955

LS	*π*	0.00316	0.02342	0.00312	0.01036	0.00017	0.00769	0.00214	0.00549
	*α*	0.04346	0.41164	0.01518	0.14545	0.02383	0.11262	0.01599	0.08213

WLS	*π*	0.00460	0.01965	0.00285	0.00841	0.00400	0.00639	0.00218	0.00458
	*α*	0.06377	0.33831	0.03373	0.11995	0.03528	0.09030	0.02999	0.06473

CVM	*π*	0.03121	0.02676	0.01369	0.01105	0.01102	0.00805	0.00618	0.00565
	*α*	0.18832	0.53935	0.08282	0.16662	0.06867	0.12406	0.04912	0.08822

AD	*π*	0.00699	0.01794	0.00358	0.00820	0.00324	0.00607	0.00151	0.00448
	*α*	0.06798	0.29733	0.03645	0.11535	0.03188	0.08419	0.02704	0.06226

RTAD	*π*	0.01797	0.02387	0.00800	0.00968	0.00743	0.00767	0.00327	0.00546
	*α*	0.16137	0.57114	0.07328	0.18995	0.06325	0.14036	0.04369	0.09924

BE${}_{SEL}$	*π*	0.19059	0.05263	0.23630	0.06427	0.25724	0.07219	0.26747	0.07603
	*α*	0.80037	0.92557	1.05120	1.27202	1.16679	1.49260	1.22156	1.59797

BE${}_{Linex1}$	*π*	0.19460	0.05441	0.23882	0.06554	0.25910	0.07319	0.26894	0.07684
	*α*	0.88038	1.10105	1.10896	1.41366	1.21176	1.60936	1.25794	1.69485

BE${}_{Linex2}$	*π*	0.18662	0.05092	0.23379	0.06301	0.25539	0.07120	0.26601	0.07522
	*α*	0.72796	0.78522	0.99720	1.14811	1.12408	1.38660	1.18668	1.50827

BE${}_{GEL1}$	*π*	0.18616	0.05089	0.23363	0.06298	0.25531	0.07118	0.26596	0.07521
	*α*	0.77466	0.87907	1.03350	1.23253	1.15330	1.45976	1.21074	1.57058

BE${}_{GEL2}$	*π*	0.17731	0.04754	0.22828	0.06045	0.25144	0.06919	0.26294	0.07359
	*α*	0.72398	0.79247	0.99840	1.15641	1.12647	1.39573	1.18920	1.51684

**Table 5:** Confidence intervals for MLEs and credible intervals for BE${}_{SEL}$, BE${}_{Linex1}$, BE${}_{Linex2}$, BE${}_{GEL1}$ & BE${}_{GEL2}$.

	**Table:** Lower ML	**Table:** Upper ML	**Table:** Lower BE${}_{SEL}$	**Table:** Upper BE${}_{SEL}$	**Table:** Lower BE${}_{Linex1}$	**Table:** Upper BE${}_{Linex1}$	**Table:** Lower BE${}_{Linex2}$	**Table:** Upper BE${}_{Linex2}$	**Table:** Lower BE${}_{GEL1}$	**Table:** Upper BE${}_{GEL1}$	**Table:** Lower BE${}_{GEL2}$	**Table:** Upper BE${}_{GEL2}$
*π* = 0.7	0.67893	1.22537	0.54643	0.78097	1.18855	0.40758	0.82882	1.16110	0.33228	0.85133	1.13581	0.28449
*α* = 1.5	1.43269	2.99595	1.56326	1.72400	2.89123	1.16723	1.89821	2.87473	0.97652	1.94425	2.79426	0.85001

*π* = 0.5	0.49112	0.88732	0.39619	0.54753	0.83908	0.29156	0.59465	0.83325	0.23860	0.62552	0.82952	0.20400
*α* = 1.5	1.44156	3.01651	1.57495	1.78700	2.96039	1.17339	1.91498	2.89365	0.97867	1.95475	2.81869	0.86394

*π* = 0.5	0.47831	0.82507	0.34676	0.55337	0.81193	0.25856	0.58375	0.80079	0.21705	0.59998	0.78400	0.18402
*α* = 2.0	1.92970	3.94662	2.01692	2.36120	3.96980	1.60860	2.56926	3.93446	1.36520	2.61150	3.83433	1.22283

*π* = 0.7	0.66515	1.14457	0.47942	0.75972	1.11726	0.35754	0.82225	1.12424	0.30199	0.83757	1.09592	0.25836
*α* = 2.0	1.91670	3.93846	2.02176	2.36761	3.94964	1.58203	2.54818	3.90039	1.35221	2.59730	3.81564	1.21834

**Figure 8:**
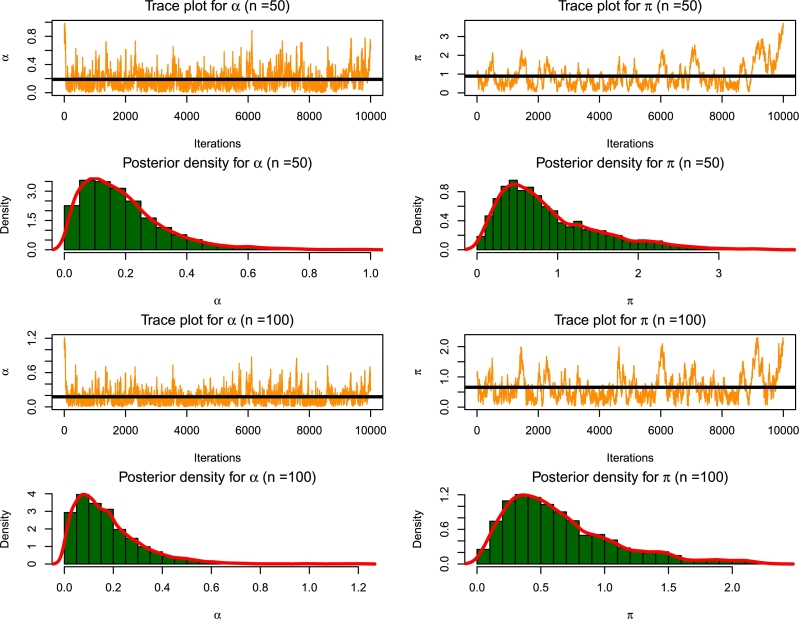
MCMC diagnostics plots for *n* = 50,100.

**Figure 9:**
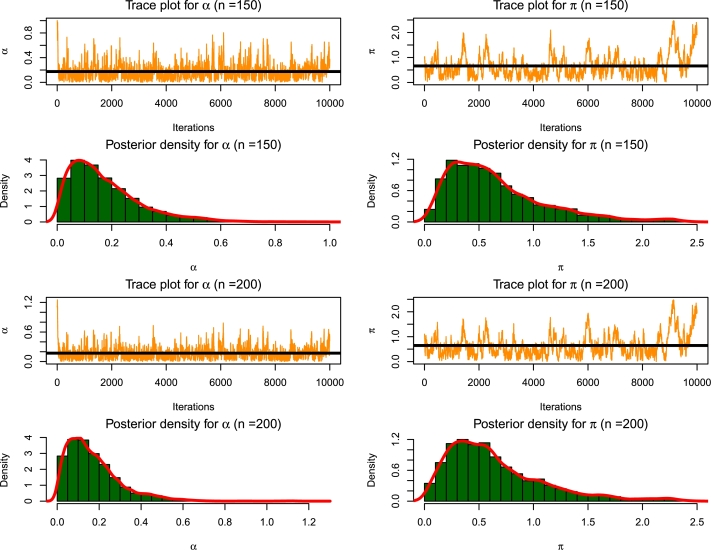
MCMC diagnostics plots for *n* = 150,200.

Figure 8, Figure 9 represent the Trace plot and posterior density plot of the parameters for varying sample sizes obtained from the simulations.

## 6 TL-BHE regression model

We begin the construction of the parametric regression model for the proposed TL-BHE by defining Y=log⁡X, where *X*∼ TL-BHE (π,α). Suppose also that $\pi =e^{-\frac{\mu }{\sigma }}$ and $\alpha =\frac{1}{\sigma }$, then the density of Log TL-BHE written as LTL-BHE for y∈ℜ is contained in equation (56)

(56) $$f(y;\mu ,\sigma )=\frac{2}{\sigma }e^{\frac{\sigma y-\mu }{\sigma }}e^{-2e^{\frac{y-\mu }{\sigma }}}\frac{\left(2+e^{\frac{y-\mu }{\sigma }}\right)}{{\left(1+e^{\frac{y-\mu }{\sigma }}\right)}^{3}}{\left\{1-{\left(\frac{e^{-e^{\frac{y-\mu }{\sigma }}}}{1+e^{\frac{y-\mu }{\sigma }}}\right)}^{2}\right\}}^{\frac{1}{\sigma }-1}$$

provided σ>0 and μ∈ℜ. The corresponding CDF is in equation (57)

(57) $$F(y;\mu ,\sigma )={\left\{1-{\left(\frac{e^{-e^{\frac{y-\mu }{\sigma }}}}{1+e^{\frac{y-\mu }{\sigma }}}\right)}^{2}\right\}}^{\frac{1}{\sigma }};\;y>0$$

The normalized density is obtained by taking $z=\frac{y-\mu }{\sigma }$, which gives equation (58)

(58) $$f(z;\mu ,\sigma )=\frac{2}{\sigma }C(z)e^{-2e^{z}}\frac{\left(2+e^{z}\right)}{{\left(1+e^{z}\right)}^{3}}{\left\{1-{\left(\frac{e^{-e^{z}}}{1+e^{z}}\right)}^{2}\right\}}^{\frac{1}{\sigma }-1}$$

where $C(z)=e^{\frac{\sigma^{2}z+\sigma \mu -\mu }{\sigma }}$. The associated survival function is in equation (59)

(59) $$S(z;\mu ,\sigma )=1-{\left\{1-{\left(\frac{e^{-e^{z}}}{1+e^{z}}\right)}^{2}\right\}}^{\frac{1}{\sigma }}$$

Using (58), we derive a regression model for the TL-BHE distribution for the variable $Y_{i}$ over a vector of covariates $v_{i}^{\prime }=(v_{i1},v_{i2},...,v_{ip})$ as in equation (60)

(60) $$y_{i}=v^{\prime }\beta +\sigma z_{i},\;i=1,2,...,n$$

provided $\mu_{i}=v^{\prime }\beta$, $\beta ={\left(\beta_{1},\cdots ,\beta_{p}\right)}^{\prime }$ is the vector of the regression coefficients and *z* is the error term with density in (58). Again, we define the survival function and density function of $Y_{i}|v^{\prime }$ in equations (61) and (62)

(61) $$S(y|v^{\prime })=1-{\left\{1-{\left(\frac{e^{-e_{i}^{z}}}{1+e_{i}^{z}}\right)}^{2}\right\}}^{\frac{1}{\sigma }}$$

and

(62) $$f(y|v^{\prime })=\frac{2}{\sigma }C(z_{i})e^{-2e_{i}^{z}}\frac{\left(2+e_{i}^{z}\right)}{{\left(1+e_{i}^{z}\right)}^{3}}{\left\{1-{\left(\frac{e^{-e_{i}^{z}}}{1+e^{z}}\right)}^{2}\right\}}^{\frac{1}{\sigma }-1}$$

where $C(z)=e^{\frac{\sigma^{2}z_{i}+\sigma \mu -\mu }{\sigma }}$ and $z_{i}=\frac{y_{i}-\mu_{i}}{\sigma }$

### 6.1 Maximum likelihood under censored data

The parameters in (62) can be estimated for right-censored data. We first define $Y_{i}$ and $C_{i}$ as the lifetime and non-informative censoring time (assuming independence) and $y_{i}=min(Y_{i},C_{i})$. Therefore, we calculate the log-likelihood for $\xi ={\left(\sigma ,\beta^{T}\right)}^{T}$ as presented in equation (63)

(63) $$\ell (\xi )=d\left[\log(2)-\log(\sigma )\right]+\sum_{i\in F}\log\left[C(z_{i})\right]-2\sum_{i\in F}e^{z_{i}}+\sum_{i\in F}\log\left(2+e_{i}^{z}\right)-3\sum_{i\in F}\log\left(1+e_{i}^{z}\right)+\sum_{i\in C}\left[1-{\left\{1-{\left(\frac{e^{-e_{i}^{z}}}{1+e_{i}^{z}}\right)}^{2}\right\}}^{\frac{1}{\sigma }}\right]$$

where *F* and *C* are the sets of uncensored and censored observations respectively and *d* is the number of failures. The MLE $\overset{ˆ}{\xi }$ of the unknown parameter vector can be obtained by maximizing (63). The accuracy of the MLEs in the new regression model is investigated from *N* samples. The algorithm for generating random samples using the acceptance-rejection method is as follows:

1. Generate *t* from the density $h(t)=\delta \beta t^{\delta -1}e^{-\beta t^{\delta }}$.
2. Generate u∼Uniform(0,1).
3. If $u\leq \frac{f(t)}{Nh(t)}$, set x=t, where f(⋅) is the PDF given in equation (6), h(t) is the PDF of the classical gamma random variable *t* and $N=\max\left[\frac{f(t)}{h(t)}\right]$. Otherwise, return to step 1.

## 7 Application to baseline CD4 count of HIV/AIDS patients records

CD4 count of individuals diagnosed of HIV/AIDS at St. Luke's Hospital, Anua, Uyo, Nigeria were collected from 2008 to 2017, see Udofia et al. [52]. The records include age, gender, date of initiation of HAART, prevalence of opportunistic infections (OIs), date of diagnostic of first opportunistic infection, baseline CD4 count, WHO HIV stage, deaths, time of follow-up. A total of 221 patients were captured in the study, and 137 of them died, and 84 were alive. The response variable $y_{i}$ represents the baseline CD4 Count. Other variables include: $\delta_{i}$: censoring indicator (0=censored,1=observedlifetime), $v_{i1}$: age (in years), $v_{i2}$: WHO state, $v_{i3}$: number of opportunistic infections, and $v_{i4}$: gender (0=male,1=female) for (i=1,⋯,221). The TL-BHE regression model for these HIV/AIDS data is written as

(64) $$y_{i}=\beta_{0}+\beta_{1}v_{i1}+\beta_{2}v_{i2}+\beta_{3}v_{i3}+\beta_{4}v_{i4}+\sigma z_{i};\;i=1,\cdots ,221,$$

where $z_{i}∼$ the PDF in equation (20).

**Figure 10:**
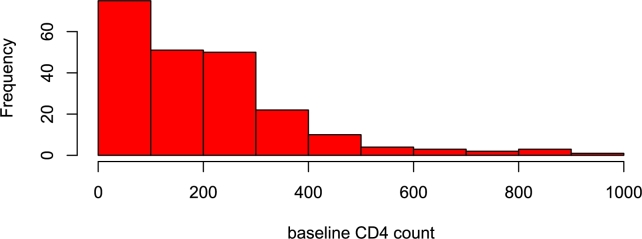
Histogram.

**Figure 11:**
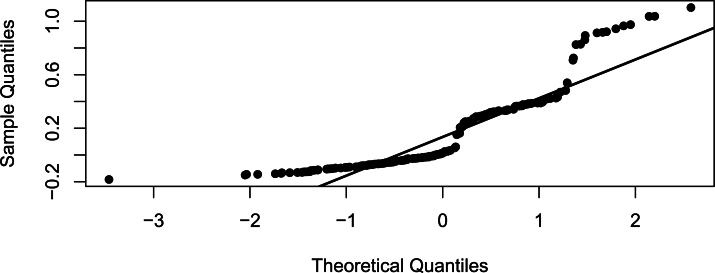
Q-Q plot.

**Figure 12:**
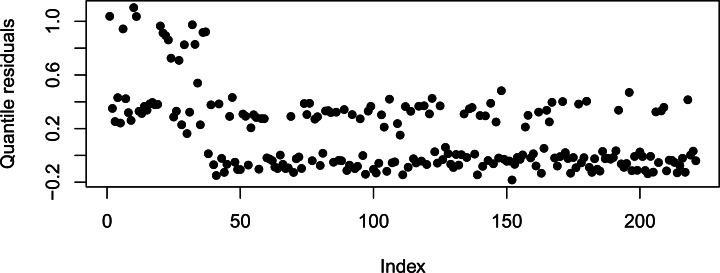
Quantile residual plot.

Figure 10, Figure 11, Figure 12 are the histogram, Q-Q plot and Quantile residual plots of the HIV/AIDS data.

In Table 6, the values in parentheses are the standard errors while values in square brackets are the p-values. The competing regression models to the proposed Log-Topp-Leone Burr-Hatke Exponential (LTL-BHE) model are the Log-Exponentiated Power Chris-Jerry (LEPCJ) by Obulezi et al. [36], Log-Power Lindley (LPL) by Ghitany et al. [24], Log-Exponentiated Power Lindley (LEPL) by Ashour and Eltehiwy [8], Log-Exponentiated Frechet (LEF) by Nadarajah and Kotz [35], Log-Power Lomax (LPLo) by Rady et al. [39], Log-Exponentiated Power Ishita (LEPI) by Ferreira and Cordeiro [22] and Log-Weibull (LW) by Weibull [54]. The NAN standard error values for parameter estimates of the EF model are because EF is sensitive to outliers. However, this sensitivity has been overcomed by the LEF since the regression parameters estimates all have their standard errors. The parametric regression model suggested from TL-BHE distribution based on the HIV/AIDS data is given as follows;

$$\text{CD4}\_{\text{count}}_{i}=5.43936+11.38415{\text{age}}_{i}-0.00286\text{WHO}\_{\text{stage}}_{i}-0.82227\text{no}\_\text{opportunistic}\_{\text{infections}}_{i}+0.0.79267{\text{gender}}_{i}+8.71427z_{i};\;i=1,\cdots ,221.$$

It indicates that only the opportunistic infections (OIs) are significant in the model among other competing variables since its p-value 0.00025 is less than 0.05 (which is the level of significance). Table 7 shows some metrics of the adequacy of the fitted regression models. The log-transformed TL-BHE regression model is more adequate for the HIV/AIDS data as it has the minimum criteria compared to the others.

**Table 6:** Regression coefficients for the HIV/AIDS patients records.

Dist	*c*	*σ*	*β*_0_	*β*_1_	*β*_2_	*β*_3_	*β*_4_
LTL-BHE	-	5.43936	18.38415	-0.00286	-0.82227	0.79267	8.71427
		(0.45290)	(8.94883)	(0.11620)	(2.41925)	(1.54788)	(2.34380)
				[0.98041]	[0.73426]	[0.60909]	[0.00025]
LEPCJ	29.05819	4.85014	-3.47376	-0.00281	-0.35644	-0.04778	0.68457
	(43.73357)	(1.73815)	(4.40202)	(0.00646)	(0.11650)	(0.07516)	(0.12361)
				[0.66373]	[0.00249]	[0.52568]	[8.64098 × 10^−8^]
LPL	-	1.32554	4.80280	0.00079	-0.29359	-0.07871	0.43700
		(0.07520)	(0.44120)	(0.00564)	(0.11575)	(0.07469)	(0.11007)
				[0.88831]	[0.01189]	[0.29311]	[9.71649 × 10^−5^]
LEPL	25.05654	4.14170	-0.91353	-0.00270	-0.35795	-0.04870	0.69204
	(39.55991)	(1.65791)	(3.82266)	(0.00656)	(0.11860)	(0.07612)	(0.12597)
				[0.68090]	[0.00285]	[0.02301]	[1.07813 × 10^−7^]
LEF	153.9891	5.15633	14.09388	0.00035	-0.34050	-0.05319	0.70967
	NAN	NAN	NAN	(0.00663)	(0.13214)	(0.08559)	(0.12773)
				[0.95767]	[0.01062]	[0.53493]	[7.90717 × 10^−8^]
LPLo	0.65350	0.59057	-5.23182	0.00179	0.41740	0.06454	-0.72328
	(0.27380)	(0.08926)	(0.59342)	(0.00708)	(0.13260)	(0.08512)	(0.13899)
				[0.80018]	[0.00187]	[0.44913]	[4.45396 × 10^−7^]
LEPI	31.92068	4.35430	-2.19554	-0.00242	-0.27263	-0.03440	0.51221
	(42.66031)	(1.02269)	(2.39576)	0.00474	(0.08592)	0.05587	(0.09324)
				[0.61031]	[0.00172]	[0.53876]	[1.0796 × 10^−7^]
LW	-	0.92439	5.60288	0.00258	-0.27031	-0.04512	0.64303
		(0.0281)	(0.47052)	(0.00609)	(0.13009)	(0.08420)	(0.11930)
				[0.67192]	[0.03887]	[0.59254]	[1.802 × 10^−7^]

**Table 7:** Performance measures for the HIV/AIDS patients records.

Dist	AIC	CAIC	BIC	HQIC	Rank
LTL-BHE	-278.8792	-278.1999	-258.40902	-270.6464	1
LEPCJ	547.5428	548.3959	571.33	557.1476	4
LPL	433.9271	434.6064	454.3161	442.1598	2
LEPL	547.5504	548.4035	571.3375	557.1552	5
LEF	550.8502	551.7033	574.6373	560.4550	6
LPLo	551.6780	552.5311	575.4651	561.2828	7
LEPI	547.0477	547.9008	570.8349	556.6525	3
LW	560.6399	561.3192	581.0289	568.8726	8

## 8 Demonstration of applicability of TL-BHE using real life data

To demonstrate the significance and flexibility of the Topp-Leone Burr Hatke Exponential (TL-BHE) distribution and showcase its competitiveness with other distribution applications, two data sets are utilized. The competing models under consideration include the following: Burr Hatke Exponential (BHE) distribution (Yadav et al. [55]), two parameters Burr Hatke exponential (2BHE) distribution (Gao and Gui [23]), Weibull (W) distribution, and exponential (Exp) distribution. Data set 1 in Table 8 is on the strength of fibres.

**Table 8:** Strengths of glass fibers (Mahmoud and Mandouh [31]).

1.014, 1.081, 1.082, 1.185, 1.223, 1.248, 1.267, 1.271, 1.272, 1.275, 1.276, 1.278, 1.286, 1.288,
1.292, 1.304, 1.306, 1.355, 1.361, 1.364, 1.379, 1.409, 1.426, 1.459, 1.460, 1.476, 1.481, 1.484,
1.501, 1.506, 1.524, 1.526, 1.535, 1.541, 1.568, 1.579, 1.581, 1.591, 1.593, 1.602, 1.666, 1.670,
1.684, 1.691, 1.704, 1.731, 1.735, 1.747, 1.748, 1.757, 1.800, 1.806, 1.867, 1.876, 1.878, 1.910,
1.916, 1.972, 2.012, 2.456, 2.592, 3.197, and 4.121

**Figure 13:**
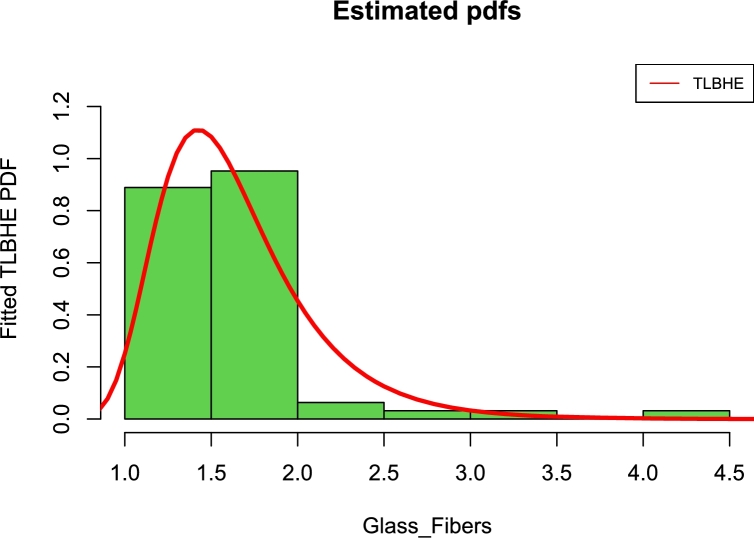
Fitted pdfs for strengths of glass fibers.

**Figure 14:**
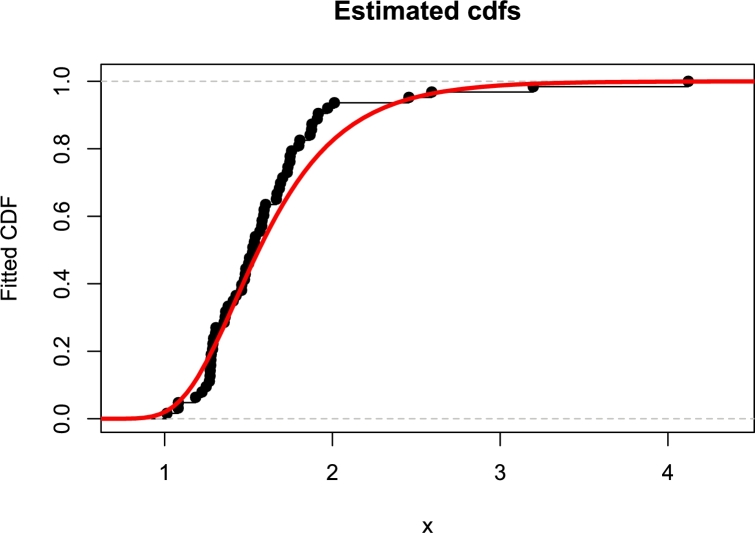
Fitted cdfs of strengths of glass fibers.

Figure 13, Figure 14 are the kernel density superimposed on the histogram and the estimated and empirical CDF for the strength of fibre data.

Data set 2 in Table 10 is on the bladder Cancer data of Patients in a hospital. It was studied by Lee and Wang [30].

**Figure 15:**
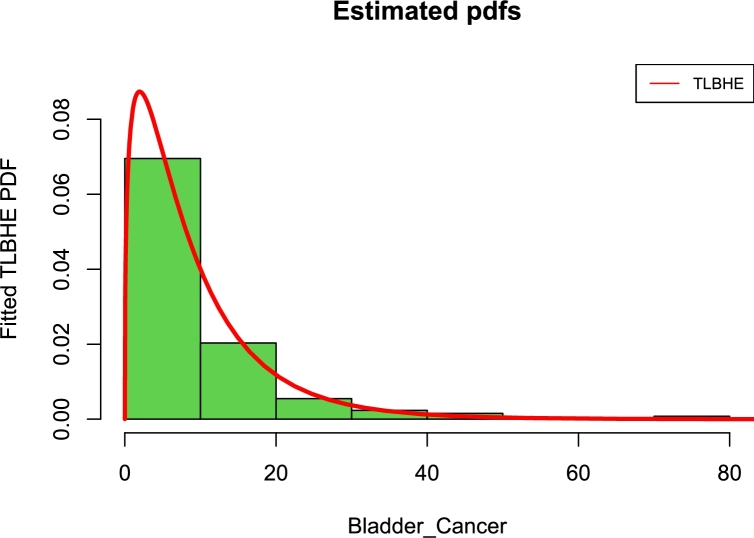
Fitted pdfs of bladder cancer patients.

**Figure 16:**
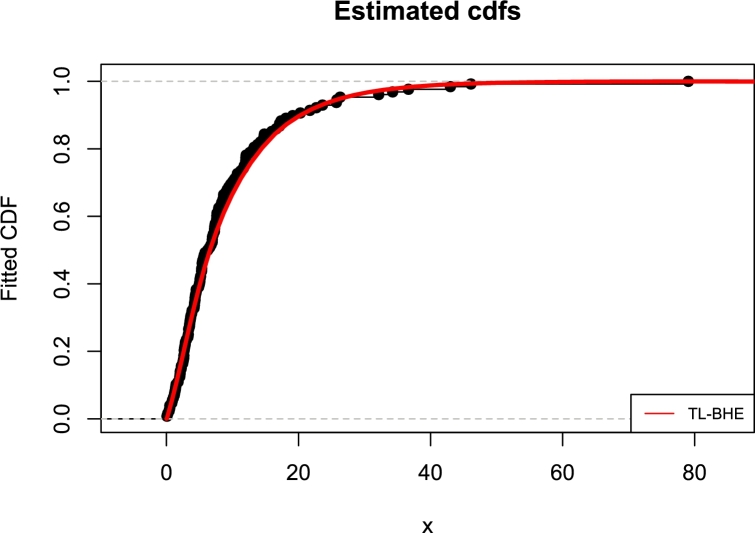
Fitted cdfs of bladder cancer patients.

Figure 15, Figure 16 are the kernel density superimposed on a histogram and the empirical and estimated CDF of the Bladder Cancer Patients data. Four distributions have been fitted as shown in Table 9 and Table 11, the estimates (Est.), Standard error (SE), log-likelihood (LL), information criteria, and goodness-of-fit statistics are presented. It is observed that the proposed model performed marginally better than other models using the two real data sets.

**Table 9:** Estimate of parameters of TL-BHE distribution and other competing models for strengths of glass fibers.

Model		Est.	SE	LL	AIC	CAIC	BIC	HQIC	CvM	AD	KS	P-Value
*TL* − *BHE*	*α*	1.075	0.1073	23.376	50.7521	50.9521	55.0383	52.4379	0.1013	0.8145	0.1240	0.2645
	*π*	142.4451	59.1514									
*TPBHE*	*θ*	0.0139	0.0177	93.5433	191.0866	191.2866	195.3729	192.3729	0.3010	2.0819	0.4728	0.001
	*α*	22.0729	28.2308									
*BHE*	*π*	0.3853	0.0525	129.4509	260.9018	260.9674	263.045	261.7447	0.2731	1.1957	0.5186	0.0.001
*W*	*θ*	0.1688	0.0379	46.3668	96.7337	96.9337	101.02	98.4195	0.7977	4.3253	0.2051	0.0084
	*β*	3.0620	0.02403									
*Exp*	*λ*	0.6189	0.0779	93.229	188.446	188.511	190.589	189.289	18.003	3.8455	0.4721	0.001

**Table 10:** Bladder cancer patients data.

0.08, 2.09, 3.48, 4.87, 6.94, 8.66, 13.11, 23.63, 0.20,2.23, 3.52, 4.98, 6.97, 9.02, 13.29, 0.40,
2.26, 3.57, 5.06, 7.09, 9.22, 13.80, 25.74, 0.50, 2.46, 3.64, 5.09, 7.26, 9.47, 14.24, 25.82, 0.51,
2.54, 3.70, 5.17, 5.41,7.62, 10.75, 16.62, 43.01, 1.19, 2.75, 4.26, 5.41, 7.63,17.12, 46.12, 1.26,
2.83, 4.33, 5.49, 7.66, 11.25, 17.14, 79.05,1.35, 2.87, 5.62, 7.87, 11.64, 17.36, 1.40,3.02, 4.34,
5.71, 7.28, 9.74, 14.76, 26.31, 0.81, 2.62, 3.82, 5.32, 7.32, 10.06, 14.77, 32.15, 2.64, 3.88, 5.32,
7.39,10.34, 14.83, 34.26, 0.90 , 2.69, 4.18,5.34, 7.59, 10.66,15.96, 36.66, 1.05, 2.69, 4.23, 7.93,
11.79, 18.10, 1.46,4.40, 5.85, 8.26, 11.98, 19.13, 1.76, 3.25, 4.50, 6.25, 8.37,12.02,2.02,3.31,
4.51, 6.54, 8.53, 12.03, 20.28, 2.02, 3.36,6.76, 12.07, 21.73, 2.07, 3.36, 6.93, 8.65,12.63,22.69

**Table 11:** Estimate of parameters of TL-BHE distribution and other competing models for bladder cancer patients data.

Model		Est.	SE	LL	AIC	CAIC	BIC	HQIC	CvM	AD	KS	P-Value
*TL* − *BHE*	*α*	0.0366	0.0044	411.363	826.7259	826.8219	832.43	829.0435	0.0678	0.4152	0.0860	0.8164
	*π*	1.3639	0.1760									
*TPBHE*	*θ*	13.5707	5.9137	415.7325	835.4649	835.5609	841.169	837.7825	0.7459	4.7187	0.0993	0.1596
	*α*	0.0076	0.0031									
*BHE*	*π*	0.0715	0.0068	480.6564	963.3129	963.3446	966.1649	964.4717	0.0724	0.4447	0.1345	0.0.01947
*W*	*θ*	0.0939	0.0190	414.0869	832.1738	832.2698	837.8778	834.4913	0.13313	0.7863	0.0700	0.5572
	*β*	1.0477	0.0675									
*Exp*	*λ*	0.1067	0.0091	414.3419	830.6838	830.7155	833.5358	831.8426	0.1192	0.7159	0.0846	0.3183

## 9 Conclusion

A new TL-BHE lifetime model has been introduced and studied. A new shape parameter is initiated to improve the flexibility of the one-parameter BH distribution. Mathematical properties like the quantile function, moments, Moment generating function, characteristic function, entropy, stress strength reliability, and order statistics have been investigated. TL-BHE model can be considered suitable for modeling left-skewed, right-skewed, approximately symmetric, and exponential data sets. Seven classical non-Bayesian parameter estimation methods were considered. An exhaustive simulation was executed by the Newton-Raphson algorithm using different sample sizes at various values of the parameters. The estimates improved as the sample size increased, and the Bias and RMSE decayed as the sample size increased. Besides, the Bayesian estimation method was studied. To test the potentiality applicability of the TL-BHE distribution, two real data sets; stress strength of glass fibers and bladder cancer patients data have been applied. From the results of the analyses, it can be easily deduced that the proposed model performed better than others using the goodness-of-fit test and information criteria. Consequently, these results can be extended since Glass fibers are widely used in various applications due to their impressive mechanical properties and performance characteristics. The strengths of glass fibers, including high tensile strength, low density, corrosion resistance, high stiffness, thermal stability, impact resistance, electrical insulation, cost-effectiveness, ease of processing, and versatility, make them an attractive choice for a wide range of industrial and commercial applications. CD4 cells, also known as T-helper cells, are a type of white blood cell that plays a crucial role in the immune system. They are responsible for signaling other immune cells to respond to infections. The CD4 count is an essential biomarker in HIV/AIDS management and research. Its applicability in various modeling approaches—ranging from disease progression and survival analysis to treatment response and population-level projections—makes it a powerful tool for understanding the dynamics of HIV infection and improving patient care. Models that incorporate CD4 count data are invaluable in guiding clinical decisions, optimizing treatment strategies, and shaping public health policies.

## 10 Further study

Further work on the proposed TL-BHE distribution can be carried out to extend the model to a bivariate case with more applications in biomedical sciences and engineering. Research can also be carried out to investigate tumor growth and time to death of Cancer patients using the TL-BHE distribution.

## CRediT authorship contribution statement

**Kizito E. Anyiam:** Writing – review & editing, Writing – original draft, Visualization, Methodology, Formal analysis, Data curation, Conceptualization. **Fatimah M. Alghamdi:** Validation, Supervision, Resources, Project administration, Investigation, Funding acquisition. **Chrysogonus C. Nwaigwe:** Validation, Supervision, Resources, Project administration, Investigation. **Hassan M. Aljohani:** Validation, Supervision, Resources, Investigation, Funding acquisition. **Okechukwu J. Obulezi:** Writing – review & editing, Writing – original draft, Visualization, Software, Methodology, Formal analysis, Data curation.

## Declaration of Competing Interest

The authors declare that they have no known competing financial interests or personal relationships that could have appeared to influence the work reported in this paper.

## Data Availability

Data included in article/supplementary material is referenced in the article.
